# Implementing Dopant-Free Hole-Transporting Layers and Metal-Incorporated CsPbI_2_Br for Stable All-Inorganic Perovskite Solar Cells

**DOI:** 10.1021/acsenergylett.0c02385

**Published:** 2021-02-01

**Authors:** Sawanta S. Mali, Jyoti V. Patil, Julian A. Steele, Sachin R. Rondiya, Nelson Y. Dzade, Chang Kook Hong

**Affiliations:** † Polymer Energy Materials Laboratory, School of Advanced Chemical Engineering, Chonnam National University, Gwangju, South Korea, 61186; ‡ Optoelectronic Convergence Research Center (OCRC), Chonnam National University, Gwangju, South Korea, 61186; § cMACS, Department of Microbial and Molecular Systems, KU Leuven, 3001 Leuven, Belgium; ⊥ School of Chemistry, Cardiff University, Main Building, Park Place, Cardiff CF10 3AT, Wales, United Kingdom

## Abstract

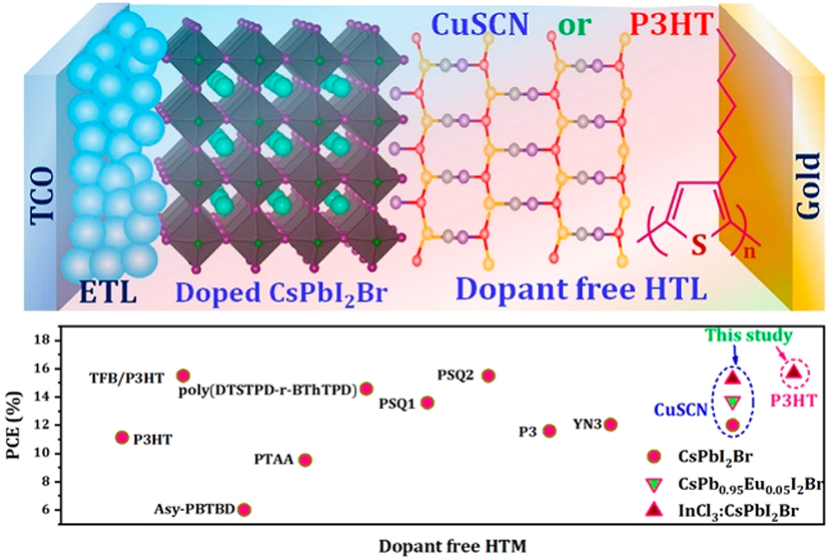

Mixed-halide CsPbI_2_Br perovskite is promising for efficient and thermally stable all-inorganic solar cells; however, the use of conventional antisolvent methods and additives-based hole-transporting layers (HTLs) currently hampers progress. Here, we have employed hot-air-assisted perovskite deposition in ambient condition to obtain high-quality photoactive CsPbI_2_Br perovskite films and have extended stable device operation using metal cation doping and dopant-free hole-transporting materials. Density functional theory calculations are used to study the structural and optoelectronic properties of the CsPbI_2_Br perovskite when it is doped with metal cations Eu^2+^ and In^3+^. We experimentally incorporated Eu^2+^ and In^3+^ metal ions into CsPbI_2_Br films and applied dopant-free copper(I) thiocyanate (CuSCN) and poly(3-hexylthiophene) (P3HT)-based materials as low-cost hole transporting layers, leading to record-high power conversion efficiencies of 15.27% and 15.69%, respectively, and a retention of >95% of the initial efficiency over 1600 h at 85 °C thermal stress.

Organic–inorganic hybrid perovskite solar cells have recently demonstrated power conversion efficiencies (PCEs) exceeding 25.5%^1^ using tunable, mixed halide and cation perovskite compositions.^2−7^ Recent works demonstrated substantial improvement in the stability of multication organic–inorganic hybrid perovskite solar cells (PSCs) through different approaches.^8−10^ However, champion devices are generally based on volatile organic cations methylammonium (MA) or formamidinium (FA) organic cations, motivating research into relatively more stable all-inorganic alternatives.^11−14^ Consequently, solar cells based on CsPbX_3_ perovskites (where X = I, Br, or Cl), and in particular iodine-rich compositions (CsPbI_3_ band gap is ∼1.7 eV), have attracted great interest.^14^ However, issues remain with regard to securing a functional CsPbI_3_-based perovskite, because of a strong tendency for its high-temperature black phases (α, β, or γ) to destabilize to an optically inactive, nonperovskite structure (δ) under ambient conditions.^15^

Photoactive β-CsPbI_3_ or γ-CsPbI_3_ phases have been stabilized at room temperature using CHI-treatment and dimethylammonium iodide (DMAI) additives, demonstrating their potential for a high PCE of 19%.^13,14^ Therefore, researchers are actively exploring different material compositions, with swapping I for relatively smaller Br halide atoms being a popular choice because of the resulting increase in the Goldschmidt tolerance factor.^16^

Utilizing mixed-halide CsPbI_2_Br perovskites is a promising avenue toward improving phase stability while retaining a solar-friendly band gap energy (*E*_g_ ≈ 1.9 eV),^17^ and they are suitable for tandem, or even triple-junction architectures.^18−20^ However, the key challenge is to synthesize device-ready CsPbI_2_Br thin films under ambient conditions without environmentally hazardous antisolvents. Reduced dimensions,^21,22^ solvent engineering strategies,^23−25^ and metal ion doping^26−32^ are promising approaches for stabilizing photoactive CsPbI_2_Br thin films, though they remain vulnerable to so-called moisture attack.^15^ Alternatively, to overcome the issues associated with ambient processing, hot-air-assisted fabrication has emerged as a compelling remedy.^33−36^

Thus far, the most efficient all-inorganic perovskite solar cells have been realized using hole-transporting layers (HTLs) doped with additives which are used to regulate hole mobility and, hence, performance. For instance, poly [bis(4-phenyl)(2,4,6-trimethylphenyl)amine] (PTAA) doped with LiTFSI (bis(trifluoromethane)sulfonamide lithium salt) and TBP (4-tertbutylpyridine) can act as efficient HTLs and have the added benefit of enhancing air stability. However, doped HTLs generally incur a high cost and increase the overall processing requirements for devices. To address this economical impairment, low-cost, dopant-free HTLs which are also hydrophobic are required.^37−39^ For the case of all-inorganic CsPbI_2_Br-based PSC, the use of poly[(dithieno[3,2-b:2′,3′-d]silolethieno[3,4-*c*]pyrrole-4,6-dione)-random-(2,2′-bithiophenethieno[3,4-*c*]pyrrole-4,6-dione)] (poly(DTSTPD-r-BThTPD)^40^ and a dopant-free donor–acceptor polymer poly(DTSTPD-r-BThTPD) have demonstrated good PCE.^41^ However, the synthesis of poly(DTSTPD-r-BThTPD) is tedious and the issue of cost still remains. Therefore, alternative HTLs such as CuSCN or P3HT are a promising choice.^37,39^ The use of cost-effective organic HTLs, like P3HT, are well-known to produce low open-circuit voltage (*V*_OC_) due to nonradiative recombination at the poorly contacting perovskite/HTL interface.^39,42^ In contrast, using highly uniform films produced via hot-air fabrication could mitigate these impairments by ensuring good contact between the layers.

Recently, Li *et al.* used P3HT for CsPbI_2_Br-based PSC and boosted the PCE from 14.08% to 15.50% using poly[(9,9-dioctylfluorenyl-2,7-diyl)-*co*-(4,4′-(N-(4-s-butylphenyl)diphenylamine)] (TFB) as a wide-band gap buffer layer.^43^ With respect to the use of low-cost, dopant-free HTLs, the highest PCEs reached thus far are 14.08% (for CsPbI_2_Br) and 11.8% PCE (CsPbI_3_) using P3HT and CuSCN, respectively.^44,45^ Because of its cost-effective nature, high thermal stability, and simplistic preparation, we have selected these promising dopant-free HTLs within the CsPbI_2_Br-based PSCs we report here.

In this work, the hot-air method is used for the fabrication of pinhole-free CsPbI_2_Br, CsPb_0.95_Eu_0.05_I_2_Br, and InCl_3_:CsPbI_2_Br perovskite thin films in order to improve their stability under ambient conditions. Highly uniform perovskite thin films facilitate excellent perovskite/HTL interfaces, enabling high-efficiency dopant-free HTL-based solar cells. Further incorporating Eu^2+^ and In^3+^ metal enhances device stability with PCEs recorded up to 15.27% and 15.69%, respectively, for CuSCN and P3HT HTLs. Importantly, these devices retain >95% of their initial PCE over 1600 h of operation in ambient conditions.

We begin by investigating the electronic band structures of the pristine and doped (Eu and In) CsPbI_2_Br materials using density functional theory (DFT) calculations (details are provided in the Supporting Information). Substituting one Pb^2+^ ion by either an Eu^2+^ or In^3+^ ion within a simulated supercell resulted in compositions CsPb_0.96_Eu_0.04_I_2_Br and CsPb_0.96_In_0.04_I_2_Br, respectively (Figure 1a–c). The lattice parameter of a CsPbI_2_Br 3 × 3 × 3 supercell is predicted to be *a* = 18.903 Å, compared to the slightly smaller lattice parameter of CsPb_0.96_Eu_0.04_I_2_Br (*a* = 18.895 Å) and CsPb_0.96_In_0.04_I_2_Br (*a* = 18.878 Å), consistent with the relative change in ionic radii (Eu^2+^, 1.12 Å; In^3+^, 0.91 Å; and Pb^2+^, 1.19 Å) in 6-fold coordination. The thermodynamic stability of Eu- and In-doped CsPbI_2_Br materials was examined by calculating the binding energy (*E*_b_) with respect to the decomposed constituent atoms using the relation
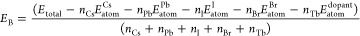
1where *E*_B_ is the binding energy, *E*_total_ the total energy of pristine CsPbI_2_Br, and *E*_atom_^A^ the energy of the free atom i (i = Cs, Pb, I, Br, and dopant Eu or In); *n*_i_ represents the number of atom i in the supercell. The binding energies of the CsPbI_2_Br, CsPb_0.96_Eu_0.04_I_2_Br, and CsPb_0.96_In_0.04_I_2_Br are predicted to be −2.68, −2.72, and −2.71 eV per atom, respectively, indicating that Eu- and In-doped materials are thermodynamically more stable than the pristine CsPbI_2_Br.

**Figure 1:**
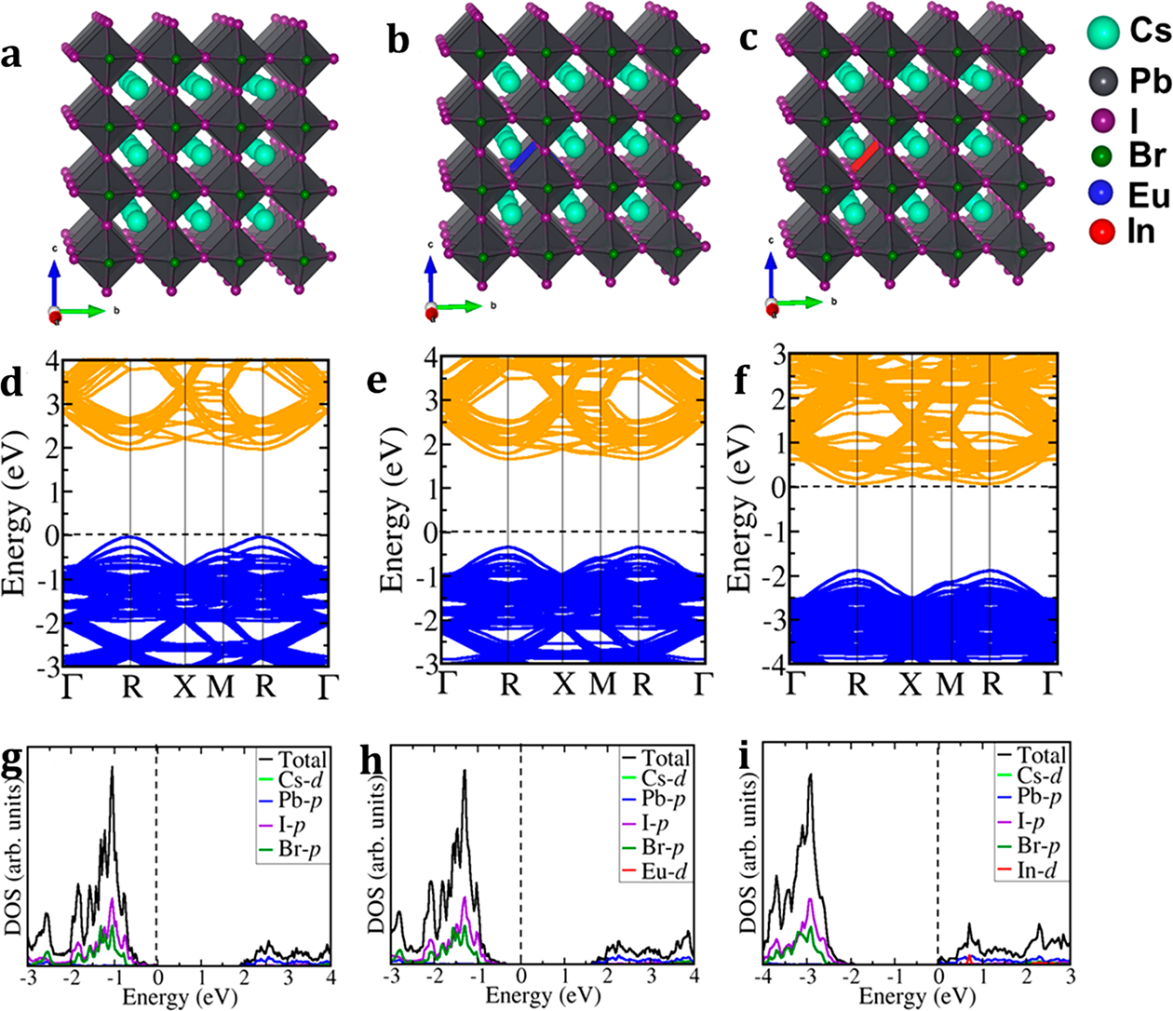
Schematic representation of the 3 × 3 × 3 supercell crystal structure of the (a) CsPbI_2_Br, (b) CsPb_0.96_Eu_0.04_I_2_Br, and (c) CsPb_0.96_In_0.04_I_2_Br and the corresponding (d–f) electronic band structures and (g–i) partial density of states.

The calculated electronic band structures (Figure 1d–f) of CsPb_0.96_Eu_0.04_I_2_Br and CsPb_0.96_In_0.04_I_2_Br also indicate direct band gap materials, with band gap energies similar to those of the parent system (∼1.9 eV). The calculated partial density of states (Figure 1g–i) show that compared to the Eu^2+^-doped CsPbI_2_Br, where the Fermi level remains closer to the valence band edge, In^3+^ incorporation shifts the Fermi level closer to the conduction band edge and introduced donor states close to the bottom of the conduction band, which may be responsible for the improved electrical conductivity of In-doped CsPbI_2_Br.^46,47^ The effective masses (m*) of holes and electrons are estimated by fitting the band edge using . The prediction of relatively small effective masses (Table S1) suggests high mobility of electrons and holes at the band edges and consequently points to efficient extraction of charge carriers in the pristine and doped CsPbI_2_Br materials.

Quality CsPbI_2_Br perovskite thin films were deposited by the hot-air method (Supporting Note 1 and Figures S1–S3). X-ray diffraction (XRD) analyses of the control CsPbI_2_Br thin film and samples doped with 5% Eu^2+^ and In^3+^ incorporation suggest only small changes in the lattice parameters. Structural refinement of the XRD data of the bare and doped CsPbI_2_Br confirms the formation of the common orthorhombic γ-phase perovskite (Figure 2a, Supporting Note 2, and Figure S4). Samples developed with InCl_3_ revealed a reduced unit cell volume, although Eu doping uncovered an expanded one, which is unexpected. Nonetheless, such structural changes suggest their incorporation into the parent lattice (further structural analysis provided below).^48^

**Figure 2:**
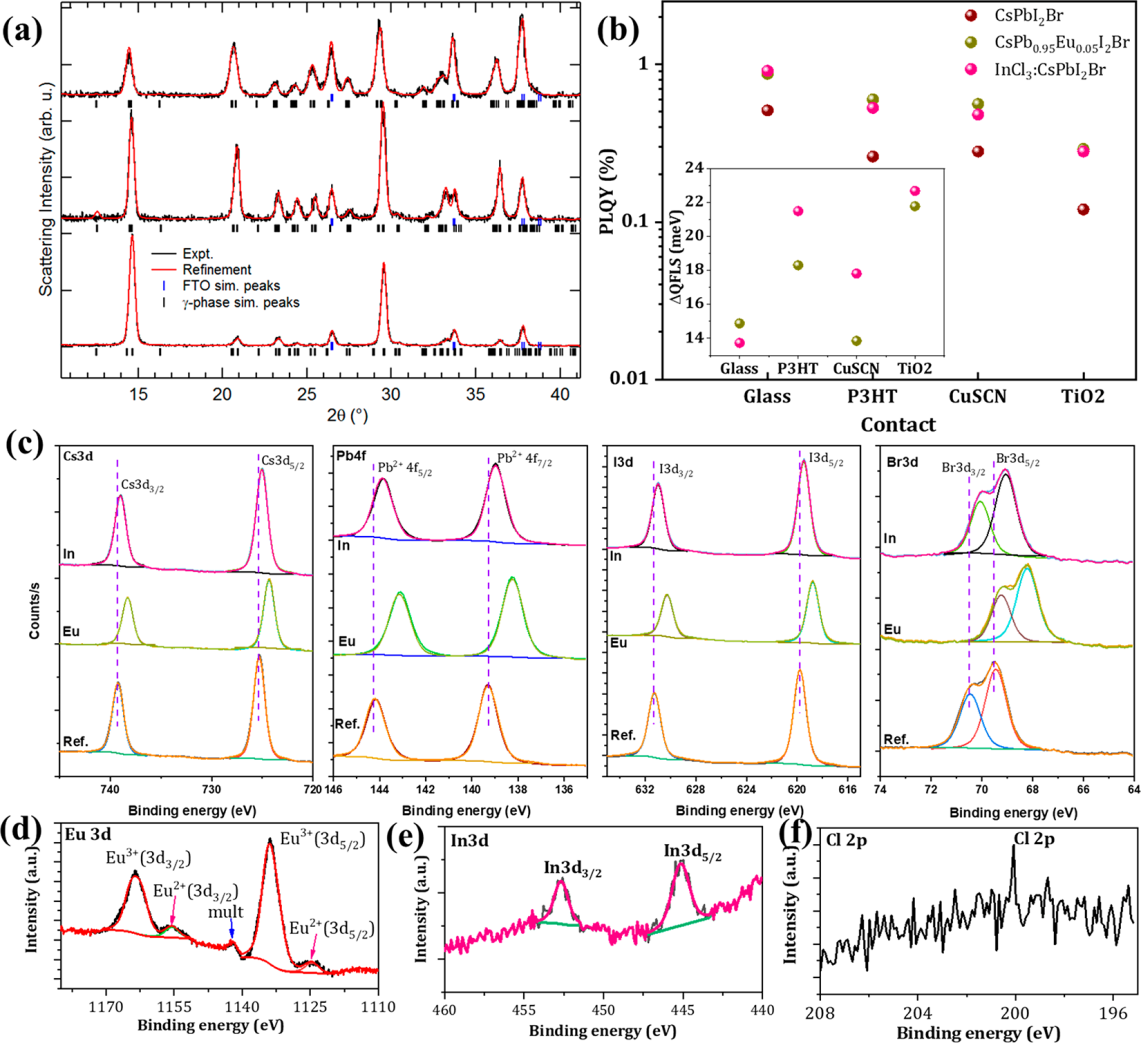
(a) Normalized XRD patterns and their structural refinements (Le Bail method) of γ-phase CsPbI_2_Br-based thin films. (b) PLQY values for CsPbI_2_Br, CsPb_0.95_Eu_0.05_I_2_Br, and 0.25% InCl_3_:CsPbI_2_Br thin films samples with no charge transport layer (glass) and with isolated p-type (CuSCN), p-type (P3HT) and n-type TiO_2_ contacts. Inset: Improvement in QFLS as determined by eq S2 for the respective samples. (c) High-resolution XPS spectra of the Cs 3d, Pb 4f, I 3d, and Br 3d core levels for CsPbI_2_Br, CsPb_0.95_Eu_0.05_I_2_Br, and 0.25% InCl_3_:CsPbI_2_Br films. (d) Eu 3d core level for CsPb_0.95_Eu_0.05_I_2_Br and (e and f) In 3d and Cl 2p core levels for 0.25% InCl_3_:CsPbI_2_Br sample. Corresponding full survey scan and peak details of the fittings can be found in Table S5 and Figures S6–S8.

We recorded time-resolved photoluminescence (TRPL) and photoluminescence quantum yield (PLQY) from the perovskite materials deposited on glass substrates and on device-relevant substrates with isolated charge transport layers (CTLs) following a previous procedure.^49,50^ With the help of PLQY analysis, we can directly observe an increase in the quasi-Fermi level splitting (QFLS) which leads to improved device performance through an increase in *V*_OC_.^51−54^ The change in the QFLS (herein ΔQFLS) for solar cell materials has been calculated previously (Supporting Note 3).^50^Figure 2b and Tables S2 and S3 show the detailed PLQY parameters of the bare and doped CsPbI_2_Br thin films in contact with HTL and ETLs. We observe improvement in the ΔPLQY values after Eu^2+^ or In^3+^ doping in the presence of both HTL and ETLs and also substantial gains for the CsPbI_2_Br thin films coated on TiO_2_ ETL, indicating nonradiative recombination in the n-i-p devices is the main factor limiting the open-circuit voltage. The PLQY is nearly 2 and 3 times larger for Eu- and In-doped films, respectively, with the expected gain of ΔQFLS = 21.78 and 22.68 meV at the TiO_2_/perovskite interface. Therefore, the doped samples are expected to exhibit enhanced *V*_OC_ once implemented in complete devices. Emission lifetimes are extracted from the fluorescence-lifetime imaging microscopic (FLIM) images in Figure S5. TRPL decay profiles (Figure S5b) were measured at ∼640 nm to examine the kinetics of photogenerated excitons and free carriers. Parameters extracted from triexponential fitting are provided in Table S4, Supporting Note 4.^55,56^ The CsPbI_2_Br, CsPb_095_Eu_0.05_I_2_Br, and 0.25% InCl_3_:CsPbI_2_Br thin film compositions exhibit extensions in average lifetime ⟨τ_avg_⟩ from 2.03 to 27 and 16 ns, respectively. The elongation of the PL decay can be ascribed to the synergetic effect of dense morphology, reduced grain boundary, compactness, limited defects, and metal ion-doped perovskite films.

Next, to investigate Eu^2+^, In^3+^, and Cl^–^ incorporation within the perovskite lattice, we performed X-ray photoelectron spectroscopy (XPS) (Figures 2c–f and S6–S8). All samples exhibited Cs 3d, Pb 4f, I 3d, and Br 3d core levels at characteristic binding energies.^57^ However, we observed that the Pb 4f, I 3d, and Br 3d peaks are shifted to lower binding energy for CsPb_0.95_Eu_0.05_I_2_Br samples, which is due to the formation of new Pb–X–Eu (X: Br and I) chemical bonding. Notably, the Cs 3d peak shows a negligible shift after Eu doping because of weak interaction between Cs^+^ and the central atom (Eu) in the octahedron (see Supporting Note 5 and Table S2) (Figure 2d).^58,59^ For the case of InCl_3_:CsPbI_2_Br, apart from the parent element signatures (*i.e.*, Cs^+^, Pb^2+^, I^–^, and Br^–^), the presence of In 3d (In 3d_5/2_, 445.16; In 3d_3/2_, 452.67 eV) and Cl 2p (200.79 eV) peaks confirms that In^3+^ and Cl^–^ are incorporated in the parent CsPbI_2_Br lattice (Figure 2e,f). Similar to the CsPb_0.95_Eu_0.05_I_2_Br sample, we have also observed a corresponding shift in the Pb 4f, I 3d, and Br 3d peaks to lower binding energies, again being ascribed to the formation of new Pb–X–In (X: Br and I) bonds. It is also noted that the shift will remain relatively small because of the small amount of doping involved (0.25% InCl_3_ doping concentration).^46^ Conversely, 2% InCl_3_:CsPbI_2_Br samples exhibited much more pronounced shifts, which evidently supports successful introduction of In (Figure S9 and Table S6.)

Furthermore, high-angle annular dark-field scanning transmission electron microscopy (HAADF-STEM) mapping of the CsPb_0.95_Eu_0.05_I_2_Br and InCl_3_:CsPbI_2_Br sample showed that the Eu^2+^ and In^3+^, along with Cl^–^, are distributed uniformly within the perovskite grains at the atomic level. In addition, the uniformity of all other Cs, Pb, I, and Br elements is seen (Figures S10 and S11). Both XPS and HAAD-STEM analysis revealed that both Eu^2+^ and In^3+^ along with Cl^–^ are incorporated evenly within the parent CsPbI_2_Br lattice.

The UV–vis optical absorption spectra of the perovskite thin films deposited on the FTO/c-TiO_2_/mp-TiO_2_ substrate are shown in Figure S12. Black-phase CsPbI_2_Br shows an absorption edge (∼640 nm/1.91 eV) which is slightly blue-shifted by the incorporation of the Eu^2+^ and nearly without shift because of In^3+^ inclusion.^32,46^ The electronic structures of the various layers were characterized by ultraviolet photoemission spectroscopy (UPS) (Figures 3a and S13). The work function (WF) and valence band maximum (VBM) were calculated from the binding energy cutoff (*E*_cutoff_) and the binding energy onset (*E*_onset_). The corresponding WF values of each layer were estimated to be 4.72, 4.68, 4.70, 4.03, and 5.08 eV for CsPbI_2_Br, CsPb_0.95_Eu_0.05_I_2_Br, InCl_3_:CsPbI_2_Br, InCl_3_:CsPbI_2_Br/P3HT, and InCl_3_:CsPbI_2_Br/CuSCN samples, respectively (Table S7). The VBM and CBM positions after Eu and InCl_3_ doping did not change drastically, while the WF shifted by 210 and 60 meV toward the CB after Eu and InCl_3_ doping, respectively. These results indicate the presence of additional negative charge carriers after Eu or In doping. Further, Eu and InCl_3_ incorporation improved the energy level alignment of the conduction band minimum between the doped CsPbI_2_Br perovskites and the ETL (Figure S14). Energy level diagrams revealed the P3HT and CuSCN, with respect to doped CsPbI_2_Br, matched favorably, resulting in efficient hole transportation. Therefore, it is expected that at the interface charge accumulation loss will be reduced and will help to improve the built-in potential across the perovskite film, resulting in higher *V*_OC_ of the doped-CsPbI_2_Br-based devices.

**Figure 3:**
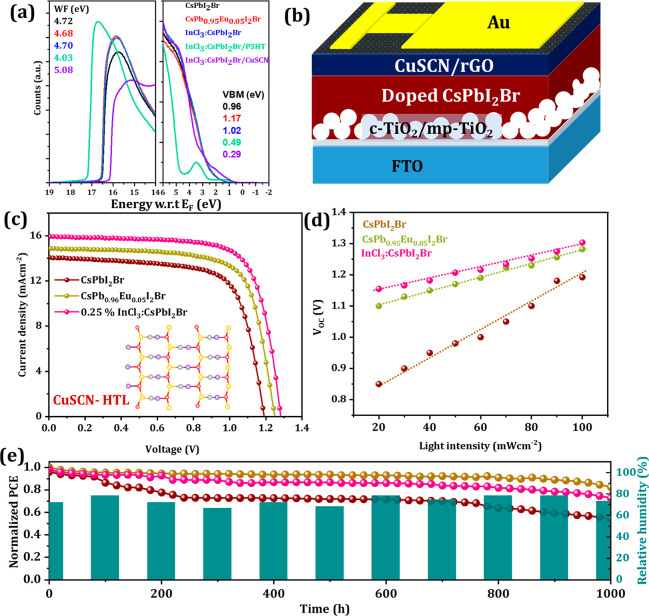
Dopant-free CuSCN HTL-based all-inorganic PSCs. (a) Ultraviolet photoelectron spectroscopy (UPS) spectra (using the He–I line with photon energy of 21.22 eV) corresponding to the secondary electron onset region (WF, work function) and valence band region (VBM, valence band minimum) of the as-prepared CsPbI_2_Br, CsPb_0.95_Eu_0.05_I_2_Br, and 0.25% InCl_3_:CsPbI_2_Br and in contact with P3HT and CuSCN (HTL) with respect to the Fermi energy (w.r.t. E_F_). VBM onsets for perovskites were determined from semilog plots (Figure S13). (b) Device architecture based on CuSCN HTL. (c) *J*–*V* characteristics. (d) Intensity-dependent *V*_OC_ variation of respective devices. (e) Normalized PCE (*for at least 5 devices for each composition*) monitored at 85 °C in ambient air conditions at 65–75% RH.

We pursue devices which have all of their layers deposited, annealed, and studied in an open atmosphere (except for gold deposition). Initially, we fabricated solar cells having the standard bilayered n-i-p device configuration, FTO/c-TiO_2_/mp-TiO_2_/CsPbI_2_Br/CuSCN/Au (Figure 3a). For comparison, we also fabricated identical devices using CsPb_0.95_Eu_0.05_I_2_Br and 0.25% InCl_3_:CsPbI_2_Br based on CuSCN HTL. The dopant-free CuSCN HTL has been deposited on annealed CsPbI_2_Br perovskite thin film by a dynamic spin coating method from 35 mg mL^–1^ DES solvent followed by rGO deposition.^60,38^Figure 3c shows the *J*–*V* characteristics of CuSCN-based CsPbI_2_Br PSCs. The cross-sectional SEM images of the doped CsPbI_2_Br thin films exhibit improved capping layer thickness from 300, 350 to 375 nm respectively for controlled, Eu^2+^ and InCl_3_-doped perovskite thin films (Figure S15). Interestingly, no grain boundaries in the cross-sectional image are observed, but rather we see the formation of a single crystalline-like layer.^61^ The perovskite device based on CsPbI_2_Br perovskite film with CuSCN HTL delivers 12.01% PCE with *V*_OC_ of 1.192 V, short-circuit current density (*J*_SC_) of 14.06 mAcm^–2^, and fill factor (FF) of 71.68%. In contrast, devices based on CsPb_0.95_Eu_0.05_I_2_Br and InCl_3_:CsPbI_2_Br have increased *V*_OC_ and *J*_SC_, because of improved thickness and film quality. The optimized devices having FTO/c-TiO_2_/mp-TiO_2_/CsPb_0.95_Eu_0.05_I_2_Br/CuSCN/Au exhibits *V*_OC_ of 1.249 V, *J*_SC_ of 14.90 mA cm^–2^, and FF of 73.76% and results in PCE of 13.72%. From cross-sectional SEM images, it is observed that both TiO_2_/perovskite and perovskite/CuSCN interfaces are highly uniform, void-free, and smooth.

Interestingly, the hot-air method resulted in a >500 nm-thick layer which is typically difficult to achieve using conventional solution processing methods. For the case of trivalent In^3+^ and Cl^–^ codoping, the *V*_OC_ is slightly higher than in the other devices and reaches 1.282 V with *J*_SC_ of 15.91 mAcm^–2^ and FF of 74.85% resulting in 15.27% PCE (Table 1). This improved performance is also reflected in the external quantum efficiency (EQE) spectra of CsPbI_2_Br, CsPb_0.95_Eu_0.05_I_2_Br, and InCl_3_:CsPbI_2_Br-based devices (Figure S16). Our champion device based on InCl_3_:CsPbI_2_Br produces ∼90% EQE values, giving *J*_int_ of 15.20 mAcm^–2^, which approaches the current values measured from *J*–*V* curves.

**Table 1:** Photovoltaic Performance Outcomes of Cells Fabricated from DHA Methods with Divalent (Eu^2+^) and Trivalent (In^3+^) Metal Ion-Doped CsPbI_2_Br All-Inorganic Perovskites Using Dopant-Free CuSCN and P3HT HTLs

sample	HTL	*V*_OC_ (V)	*J*_SC_ (mA cm^–2^)	FF (%)	PCE (%)
CsPbI_2_Br _(average)_	CuSCN	1.185	13.80	70.00	11.45
CsPbI_2_Br _(champion)_		1.192	14.06	71.68	12.01
CsPb_0.95_Eu_0.05_I_2_Br_(average)_		1.235	14.45	72.00	12.84
CsPb_0.95_Eu_0.05_I_2_Br_(champion)_		1.249	14.90	73.76	13.72
0.25% InCl_3_:CsPbI_2_Br_(average)_		1.275	15.55	74.5	14.77
0.25% InCl_3_:CsPbI_2_Br_(champion)_		1.282	15.91	74.85	15.27
					
0.25% InCl_3_:CsPbI_2_Br _(average)_	P3HT	1.295	15.65	74.50	15.09
0.25% InCl_3_:CsPbI_2_Br_(champion)_		1.303	15.90	75.76	15.69

Furthermore, the stabilized power output (SPO) of the champion devices were monitored for full sun illumination over 200 s (Figure S17). The steady-state photocurrent outputs for the CsPbI_2_Br-, CsPb_0.95_Eu_0.05_I_2_Br-, and InCl_3_:CsPbI_2_Br-based devices are 14.30, 14.63, and 15.64 mAcm^–2^, respectively, and all exhibit *J*_SC_ values close to those obtained from *J*–*V* curves. The steady-state output values yielded stabilized PCE values of 12.02%, 13.61%, and 15.10% for CsPbI_2_Br-, CsPb_0.95_Eu_0.05_I_2_Br-, and InCl_3_:CsPbI_2_Br-based devices, respectively. In contrast, the current density output of the bare CsPbI_2_Br-based devices declined continuously, whereas the CsPb_0.95_Eu_0.05_I_2_Br and InCl_3_:CsPbI_2_Br-based devices persisted far longer. This stabilized performance arises from the incorporation of metal cation doping, which stabilizes the photoactive black-phase (mechanism outlined below) and reduces the nonradiative recombination.

For charge transport measurements, we recorded the *V*_OC_ as a function of illumination intensity for the examined perovskite devices (Figure 3d). The slope of the fitted data yields the ideality factor (η), which was determined by *V*_OC_ = *nkT* ln(*I*)/*q* + *A*, where *k*, *T*, and *q* are Boltzmann constant, the temperature in Kelvin, and the elementary charge, respectively. Parameter *A* is a constant according to the Shockley–Read–Hall (SRH) recombination mechanism.^62−64^ The above equation can be simplified for η as, . It is considered that the trap-assisted recombination played a dominant role in determining the characteristics of devices at η = 2. Our controlled device gives η values as high as 1.52, which decreased to 1.23 and 1.15 for CsPb_0.95_Eu_0.05_I_2_Br and 0.25% InCl_3_:CsPbI_2_Br-based devices, respectively, indicating the suppression of charge recombination.

Next, we monitored hysteresis and found control devices exhibited PCE values of 11.09% and 12.01% for the forward and reverse scans, respectively (Figure S18). In contrast, the CsPb_0.95_Eu_0.05_I_2_Br-based device exhibited 12.51% and 13.72% PCE in forward and reverse scan which is reduced because of highly uniform film quality. Further, we have also monitored the device performance of our champion InCl_3_:CsPbI_2_Br-based devices which exhibited *J*_SC_ of 15.61 mA cm^–2^, *V*_OC_ of 1.262 V, and FF of 72.42%, resulting in PCE of 14.26% in the forward scan. On the other hand, the reverse scan exhibited 15.27% PCE with *J*_SC_ of 15.91 mA cm^–2^, *V*_OC_ of 1.282 V, and FF of 74.85%; Table S8 indicates less hysteresis than the control architecture.

We selected the best performing devices from each composition and studied the air-stability of unencapsulated devices under continuous white light LED illumination, equivalent to 100 mW cm^–2^ in an ambient condition at 85 °C thermal stress. All devices were monitored under identical conditions; however, we have not regulated the environmental monitoring/control throughout the measurements. The stability analysis of these champion devices exhibited higher device performance due to excellent thermal stability properties of CuSCN HTL (Figure 3e). The bare CsPbI_2_Br-based device maintained ∼60% initial efficiency after 1000 h; however, In^3+^ and Eu^2+^ incorporated devices retain over 75% and 87%, respectively, indicating excellent device stability.

P3HT is another low-cost HTL and can be used without any additive dopants, and we further examine its suitability for our developed champion InCl_3_:CsPbI_2_Br absorber composition (Figure 4a).^42^ These devices exhibited 14.83% (with *V*_OC_ = 1.277 V, *J*_SC_ = 15.90 mAcm^–2^, and FF = 73.10%) and 15.69% (with *V*_OC_ = 1.303 V, *J*_SC_ = 15.91 mAcm^–2^, and FF = 75.76%) for forward and reverse scans, respectively (Figure 4b and Table S9). The stability analysis revealed the P3HT devices also retain >95% of initial PCE over 1600 h, indicating excellent ambient stability under 85 °C thermal stress (Figure 4c). The photostability under 1 sun continuous illumination for several hours indicated the doped sample exhibited excellent photostability as compared to the control device (Figure 4d). The InCl_3_:CsPbI_2_Br/P3HT champion unencapsulated device was sent for third-party efficiency testing and was confirmed to exhibit a certified 14.97% PCE (see Figure S19).

**Figure 4:**
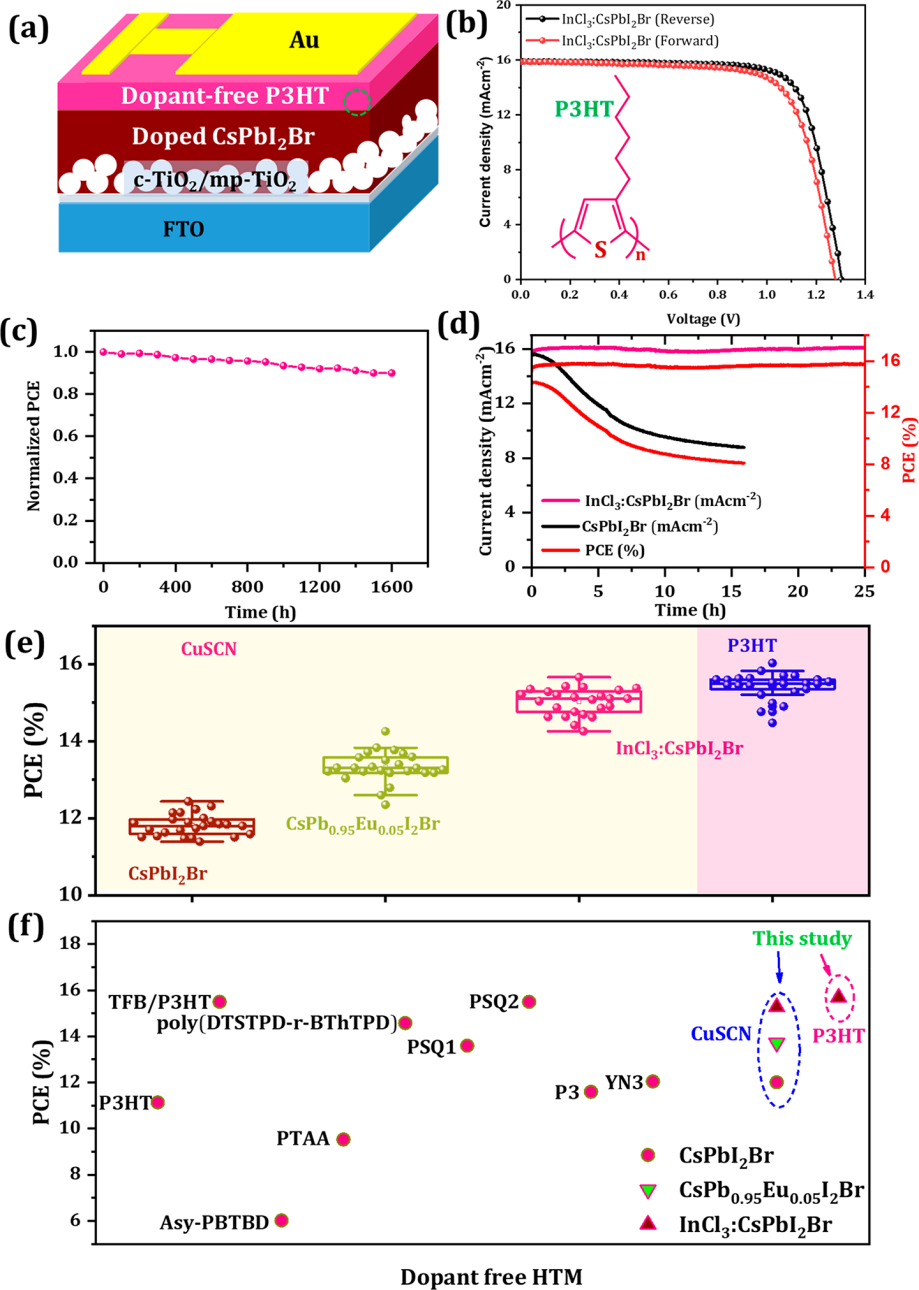
(a) Dopant-free P3HT HTL-based device configuration. (b) *J*–*V* characteristics of InCl_3_:CsPbI_2_Br-based solar cells using dopant-free P3HT. (c) Ambient conditions device stability at 40–50% RH at 85 °C thermal stress of the 5 devices. (d) Steady-state current density and PCE of unencapsulated CsPbI_2_Br and InCl_3_:CsPbI_2_Br devices monitored under continuous 1.5 AM solar-simulator illumination as a function of time. (e) Device performance distribution for dopant-free HTLs for InCl_3_:CsPbI_2_Br-based PSCs. (f) Distribution of PCE based on different dopant-free HTLs reported to date. Data has been extracted from refs (40, 43, 65, and 65).

For comparison, we have also fabricated conventional additive-doped spiro-MeOTAD-based devices and monitored their ambient stability (Figure S20). Unfortunately, even though we used expensive additive-based spiro-MeOTAD HTL, it shows lower efficiency. The best-performing device exhibited 13.70% PCE with *V*_OC_ = 1.197 V, *J*_SC_ = 15.81 mAcm^–2^, and FF = 72.42%. This may be linked to the deposition of spiro-MeOTAD-based HTL under ambient conditions and its moisture instability. The ambient stability monitored under ambient conditions revealed sharp drops up to 50% within a few hours and retained only ∼20% of the initial PCE only after 100 h. This is due to the sensitive nature of additives-doped Spiro-MeOTAD HTL. Furthermore, we fabricated dopant-free spiro-MeOTAD-based devices, but these devices showed limited PCE (Figure S21).

Figures 4e and S22 show the statistical distribution of the PCEs in forward and reverse scan based on different compositions and methods. The statistical distribution of the PCE revealed high repeatability of the device performance. Interestingly, the reproducibility of the Eu^2+^- and In^3+^-doped CsPbI_2_Br-based devices were better in comparison to those prepared with the bare CsPbI_2_Br compositions. For instance, in the CsPbI_2_Br-based devices, we observed an average PCE of ∼12.2% with *V*_OC_ of 1.18 ± 0.01 V, *J*_SC_ of 14.21 mAcm^–2^, and FF of 72 ± 2%; however, the CsPb_0.95_Eu_0.05_I_2_Br-based devices exhibited excellent reproducibility with *V*_OC_ of 1.250 ± 0.01 V, *J*_SC_ of 15.30 ± 0.5 mAcm^–2^, and FF of 73 ± 2% yielding average efficiency >13.50 ± 0.1%. As expected, all 0.25% InCl_3_:CsPbI_2_Br-based devices exhibited higher current density with *V*_OC_ exceeding 1.30 V, which is due to the synergistic effect of hot-air method, metal-ion doping, and the suitability of the HTL.

We believe that our hot-air processed devices also exhibited state-of-the-art power conversion efficiency for dopant-free HTL-based devices (Figure 4f).^65,66^ We conclude that although there are a range of deposition methods and different dopant-free HTLs which can be explored, our combination of hot-air method with low-cost, dopant-free CuSCN and P3HT HTL-based devices embodies a promising route for high-efficiency devices that can be both processed and operated under fully ambient conditions. Among the previous methods used for inorganic perovskite solar cells to date, our work showed a record PCE of 15.69% for InCl_3_:CsPbI_2_Br composition and dopant-free P3HT HTL.

The stabilizing mechanism of metal doping in the normally unstable CsPbI_2_Br parent perovskite system is yet to be discussed. Within a moisture-rich ambient atmosphere, water acts as a catalyst toward phase decay and δ-phase (nonperovskite) formation, partially dissolving the surface halide anions and introducing vacancies.^67^ As a result, the increased concentration of surface halide vacancies lowers the kinetic barrier and accelerates phase degradation, *i.e.* turning into the δ-phase. This is clearly tracked in Figures S23 and S24, showing the decay of the bare CsPbI_2_Br perovskite thin films within hours of ambient storage. A complete transformation of the perovskite into the δ-phase is confirmed via structural refinement of the XRD patterns recorded after degradation, confirming the absence of any detectable crystalline side-products. This confirmed that the degradation mechanism under an ambient atmosphere is perovskite phase destabilization. The incorporation of InCl_3_ does little to slow a similar degradation pathway in an exposed thin film (*i.e.*, no top contact layers attached); however, Eu is relatively successful in preserving the perovskite phase under the same conditions.

The change in stability cannot be accounted for purely based on a model which considers changes to the lattice tolerance factor, whereby reducing the unit cell volume (via B site doping) can increase the tolerance factor when the A and X sites remain unchanging. Similar stabilizing effects have been demonstrated recently for CsPbI_3_ doped with a few percent of Bi^3+^.^68^

For phase transitions in which the high-symmetry α-phase is reduced to a degenerate γ-phase, the number of distortion components can be expressed in terms of symmetry-adapted strains.^69,15^ The decoupled strain components are presented in Figure S25 (Supporting Note 6). Starting with a bare γ-CsPbI_2_Br perovskite, both InCl_3_ and Eu doping suppress the strain-related distortions in the perovskite crystal, making it more cubic-like (*i.e.*, α-phase). Like the stabilizing effect of heating CsPbI_2_Br toward a stable cubic perovskite structure, we suggest that the doping stabilizes the system because of a reversal of the spontaneous strains leading to phase decay. In addition, this effect is the largest for the Eu system, which we find is the most stable when used in an ambient-stable device.^70^ Furthermore, we have recorded focused ion beam (FIB) cross-sectional images of the fabricated devices using the CsPbI_2_Br-based absorbers (Figure S26). To study degradation, we have obtained FIB images of the fresh devices (∼10 h after fabrication) and after 14 days of aging. The FIB images of the freshly prepared devices exhibited formation of compact capping layers onto mp-TiO_2_ ETL. Interestingly, the P3HT layer is uniformly deposited and the perovskite/P3HT interface is smooth. In the present investigation, we have used doped CsPbI_2_Br perovskite compositions, which is free from conventional organic cations such as MAI, FAI, or DMAI. Therefore, there is less possibility of the formation of HI gases during degradation. However, we observed the formation of bright spots and dark voids in the CsPbI_2_Br-based materials, which revealed iodine degradation or halide ion-migration and the formation of δ-CsPbI_2_Br or PbI_2_.^71^ This is the most likely degradation of the bare CsPbI_2_Br-based devices. On the other hand, our CsPb_0.95_Eu_0.05_I_2_Br- and InCl_3_:CsPbI_2_Br-based devices exhibit an intact morphology of the capping layer even though we recorded them on devices aged for 14 days, indicating the devices were well-preserved.

In summary, we have utilized a simple hot-air method for the fabrication of high-quality CsPbI_2_Br thin films under ambient conditions. Our control over the deposition and the incorporation of Eu and In cations offers a new approach for stabilization of a functional CsPbI_2_Br black-phase in ambient air. The XRD, XPS, and STEM-HAADF analyses evidenced that the Eu and In cations are successfully incorporated into CsPbI_2_Br crystal which inhibits the black-to-yellow phase transformation by releasing spontaneous strains in the lattice. Our TRPL measurements revealed longer carrier life times due to Eu and In incorporation, indicating the passivation of traps. The fabricated InCl_3_:CsPbI_2_Br-based PSCs devices based on dopant-free, low-cost CuSCN and P3HT HTLs exhibited record PCEs of 15.27% and 15.69%, respectively. Long-term thermal analysis revealed more than 95% retention over 1600 h of operation under ambient conditions, which is much greater than the conventional additives-doped Spiro-MeOTAD HTL. We believe that our hot-air processed devices exhibited state-of-the-art power conversion efficiency in dopant-free HTL-based PSCs. These results provide new insights for the fabrication of high-quality cesium-based PSCs and low-cost dopant-free HTLs with excellent efficiency and air–thermal stability.

## Supporting Information Available

Full experimental procedures and device fabrication, DFT analysis details, materials, experimental methods, materials’ characterization, detailed discussion on methods and XPS analysis, PLQY calculations, TRPL analysis, HAADF-STEM, FIB, stability analysis, and testing (PDF)
