# Supplementary material for: Implementing Dopant-Free Hole-Transporting Layers and Metal-Incorporated CsPbI2Br for Stable All-Inorganic Perovskite Solar Cells
Source: ACS Energy Lett. 2021 Feb 1;6(2):778–88. doi: 10.1021/acsenergylett.0c02385 (PMC8018314; doi:10.1021/acsenergylett.0c02385)
Supplement: Supplementary file 1 — nz0c02385_si_001.pdf [file nz0c02385_si_001.pdf]

Implementing Dopant-Free Hole Transporting Layers and Metal Incorporated $CsPbI_2Br$ for Stable All-Inorganic Perovskite Solar Cells

Sawanta S. Mali,[a] Jyoti V. Patil,[a,b] Julain A. Steele,[c] Sachin R. Rondiya,[d] Nelson Y. Dzade,[d] and Chang Kook Hong*[a]*

*[a]*Polymer Energy Materials Laboratory, School of Advanced Chemical Engineering, Chonnam National University, Gwangju, South Korea-61186.*

Email: sawanta@jnu.ac.kr (SSM), hongck@jnu.ac.kr (CKH)

[b]Optoelectronic Convergence Research Center (OCRC), Chonnam National University, Gwangju, South Korea-61186.

[c]cMACS, Department of Microbial and Molecular Systems, KU Leuven, 3001 Leuven, Belgium

[d]School of Chemistry, Cardiff University, Main Building, Park Place, Cardiff, CF10 3AT, Wales, United Kingdom

Computational Details

The structure calculations were performed using the Vienna Ab initio Simulation Package (VASP), [S1-S3] using the project augmented wave (PAW) method. [S4] The electronic exchange–correlation potential was calculated using the Perdew–Burke–Ernzerhof (PBE) generalized gradient approximation (GGA) functional.[S5] For accurately prediction of the electronic structures (band structure and partial density of states), the screened hybrid functional HSE06[S6] was used with Hartree−Fock exchange value of 25%. Due to the strong relativistic correction on heavy atoms, the spin−orbit coupling (SOC) was accounted for in calculations. [S7] The cutoff energy for the plane wave functions is set to 600 eV and the threshold of the total energy change is set to 10^{-6} eV. A 3×3×3 Monkhorst−Pack[S7] *k*-point mesh of was used in geometry optimization and a higher *k*-point mesh of 5×5×5 was used for electronic structure calculations. A 3×3×3 $CsPbI_2Br$ supercell consisting of 135 atoms in total was used to characterize the Eu and In-doped $CsPbI_2Br$ systems. By substituting one Pb^{2+} ion by one Eu^{2+} or In^{3+} ion resulted in Eu- and In-doped $CsPbI_2Br$ with compositions $CsPb_{0.96}Eu_{0.04}I_2Br$ and $CsPb_{0.96}In_{0.04}I_2Br$, respectively.

Methods

Materials. All of the chemicals and materials were purchased and used without further purification. Materials including solvents Dimethyl sulfoxide (DMSO), (N, N-Dimethylformamide (DMF, Sigma Aldrich), CsI (Sigma Aldrich, 99.999 %) and $PbBr_2$ (Sigma, ≥98%), PbI_2 (Sigma Aldrich, 99%), EuI_2 (Sigma, 99.99), Indium(III) chloride ($InCl_3$) (Sigma, 98 %), poly(3-hexylthiophene-2,5-diyl) (P3HT) (Sigma-Aldrich, average Mw 85,000-100,000), Copper(I) thiocyanate (CuSCN) (>99%) and diethyl sulfide (98%) were purchased from Sigma-Aldrich and used as received. Hot-air gun (BOSCH, GHG 630 DCE Hot Air Gun - 0601 94C 740) was used as a hot-air source.

Preparation of c-TiO_2 electron transport layer (ETL). Pattern FTO-coated glass substrates (8 Ωm, iTASCO) were ultrasonically cleaned in a diluted Hellmanex soap solution, rinsed with deionized water, acetone, ethanol and then treated with UV-ozone for 15 min. Nearly 50-60 nm-thick compact TiO_2 (c-TiO_2) layer was deposited on the substrates by first spin coating the TiO_2 precursor and then annealing in air at 450 °C for 30 min. The precursor composition was as follows: 1 ml commercial titanium isopropoxide solution (Sigma-Aldrich) that was diluted in ethanol containing 12M HCl. Nearly, 180 nm mp-TiO_2 layer was composed of the 30-nm-sized particles and deposited by spin coating a commercial TiO_2 paste (Dyesol DSL-30NR-T, Dyesol) diluted in ethanol (6:1 weight ratio) at 4000 rpm for 20 s with ramp rate

2000 rpm s-1. After drying at 125 °C, the TiO_2 films were gradually heated to 500 °C, baked at this temperature for 15 min and then cooled to room temperature. The mesoporous deposited film was again treated with $TiCl_4$ followed by sintering at 450 °C for 30 min.

Synthesis of the perovskite solution

$CsPbI_2Br$ preparation. The 1.2 M $CsPbI_2Br$ perovskite precursor solution was prepared by stoichiometrically mixing 0.277 g PbI_2 (TCI), 0.220 g $PbBr_2$ (Sigma) and 0.312 g CsI (Sigma), in 1ml anhydrous DMSO. This solution was stirred overnight at 75 °C and then filtered through a 0.2 μm syringe filter.

$CsPb_{0.95}Eu_{0.05}I_2Br$ preparation. The $CsPb_{0.95}Eu_{0.05}I_2Br$ perovskite precursor was prepared by mixing desired volume of 1.2 M $CsPbI_2Br$ in 1 ml anhydrous DMSO), 1.2 M CsBr formamide solution and 1.2 M EuI_2 DMF: DMSO (v:v=4:1) solution stoichiometrically in ambient condition.

$InCl_3$:$CsPbI_2Br$ preparation. Initially 2 % $InCl_3$ power was mixed in above $CsPbI_2Br$ solution and used as a stock solution. Desired volume of 2 % $InCl_3$:$CsPbI_2Br$ solution was used for 0.25% $InCl_3$ doping.

Device fabrication. The clear filtered 50 μl yellow solution was spin-coated on the top of the FTO/c-TiO_2/mp-TiO_2 electrode by a consecutive two-step spin coating process at 1,000 and 3,000 rpm for 10 and 30 s, respectively. Experimental details are discussed in our previous report. [S8, S9]

Synthesis of CuSCN HTM:

35 mgml^{-1} CuSCN HEL in diethyl sulfide (DES) solution was prepared by continuous stirring for 30 min at room temperature. The cleared solution was filtered through syringe filter and used for deposition. For CuSCN deposition, we have used dynamic spin-coating method and CuSCN solution was dropped quickly onto $CsPbI_2Br$ thin film spinning at 5000 rpm followed by 40 sec further spinning. The CuSCN deposited samples were further dried on hot plate at 100 °C for 5 min and used for rGO deposition. 1 mgml^{-1} well dispersed reduced graphene oxide (rGO) in chlorobenzene solution was deposited by spin coating onto the CuSCN HTM at 3000 rpm for 30 sec. Then, the substrates were transferred to a vacuum chamber subsequently evacuated to a pressure of 2×10^{-6} mbar. The devices were completed by deposited 60 nm Au onto the CuSCN/rGO layer through shadow masks with an active area of 0.09 cm^2.

Preparation of P3HT based HTM

The P3HT hole transporting material was prepared by dissolving P3HT in chlorobenzene (15 mg.ml^{-1}) and used without any additive dopants. The prepared P3HT HTM solution was spin-coated on the FTO/c-TiO_2/mp-TiO_2/perovskite at 3,000 rpm for the 30 s on

preheated (100 °C) perovskite thin film quickly. Fabricated devices were further annealed at 200 °C for 5 min and then the devices were transferred to a vacuum chamber and evacuated to a pressure of 2×10^{-6} mbar. For the counter electrode, a 60 nm thick Au contacts were deposited on the top of the P3HT over layer by thermal evaporation (growth rate ~0.5Å s^{-1}). The active area of this electrode was fixed to 0.09 cm^2. An active area was calculated as per gold and laser pattern cross-sectional area. The exact illumination to the active area was fixed by attaching thin metal shadow mask from the backside during measurements.

Preparation of Spiro-MeOTAD based HTM

The spiro-OMeTAD solution was prepared by dissolving spiro-OMeTAD in chlorobenzene (72.3 mg ml^{-1}) containing 17.5 μl of a solution of Li-TFSI in acetonitrile (520 mg ml^{-1}) and 28.8 μl 4-tert-butylpyridine.

Perovskite thin film characterizations. The top-surface and cross-sectional images were recorded by a field emission scanning electron microscope (FESEM; S-4700, Hitachi). X-ray diffraction (XRD) measurements were carried out using a D/MAX Ultima III XRD spectrometer (Rigaku, Japan) with Cu Kα line of λ=1.5410 Å. A double beam spectrophotometer (Varian, CARY, 300 Conc.) in the 280–800 nm wavelength range. An inverted-type scanning confocal microscope (Picoquant, MicroTime 200, Germany) with a 100 × (oil-immersion) objective was employed to measure the fluorescence lifetime imaging (FLIM). Single-mode pulsed solid state diode laser (470 nm with ~30 ps pulse width, ~0.1 μW average power, and operated at 2.5 MHz repetition rate) was used as an excitation source. The instrumental response function of the system was ∼100 ps at FWHM. A dichroic mirror (490 DCXR, AHF), a long-pass filter (HQ500lp, AHF), a 75 μm pinhole, a band-pass filter (FB550-40, Thorlabs), and a single photon avalanche diode (PDM series, MPD) were used to collect emissions from the bare and doped $CsPbI_2Br$ samples. Time-correlated single-photon counting (TCSPC) technique was used to count the time-resolved emission signals. Typically, FLIM images of an 80 μm × 80 μm perovskite sample area consisting of 200 × 200 pixels, were recorded using the time-tagged time-resolved (TTTR) data acquisition method. The acquisition time of each pixel was 2 ms per pixel. Photoluminescence lifetime images and their exponential fittings for the obtained fluorescence decays, extracted from the FLIM images, were performed using SymPhoTime-64 software provided by the manufacturer in an exponential decay model, where I(t) is the time-dependent PL intensity, A is the amplitude, τ is the PL lifetime, and i is 3. Steady-state fluorescence spectrum 5 at a focal volume was measured for the samples by guiding the fluorescence signal to the external spectrometer (F-7000, Hitachi).

Absolute PLQY measurements

Absolute PL quantum yield spectrometer system (Hamamatsu, C9920-02/-02) was used for absolute PL Quantum Yield measurement. Absolute PL quantum yield (PLQY) measurements were performed following the procedure of de Mello and co-workers. [S10] A 405 nm laser was used to photoexcite the samples placed in an integrating sphere and a Maya pro spectrometer used to measure the signal.

XPS analysis and its fitting

A Thermo Scientific K-ALPHA$^+$ X-Ray Photoelectron spectrometer was used to perform XPS measurements using a monochromatic Al Kα X-Ray source at a take-off angle of 60 degrees. The core level XPS spectra were recorded using a pass energy of 50 eV (resolution approximately 0.4 eV) from an analysis area of 400 μm x 400 μm. The spectrometer work function and binding energy scale were calibrated using the Fermi edge and 3 d peak recorded from a polycrystalline silver (Ag) sample prior to the commencement of the experiments. Fitting procedures to extract peak positions from the XPS data were carried out using XPS peak 4.1/Magicplot. A Shirley background was used and the spectra were fit with a mixture of Gaussian/Lorentzian (Lorentzian = 30 %) line shapes.

TEM and HAADF-STEM

Transmission electron microscopy was performed on a high-resolution transmission electron microscopy (HRTEM) TECNAI F20 Philips operated at 200 KV. Energy-dispersive X-ray (EDX) elemental mapping was carried out in the scanning transmission electron microscopy (STEM) mode. The contrast against the background of HAADF-STEM image for mapping has been carried our background selection: ROIs mode at detector angle: 14.6 degree.

Focused Ion Beam (FIB)

Focused Ion Beam (FIB) System NX2000 used for analysis of cross sectional images of fabricated devices for different time interval.

Photovoltaic studies. The cells were illuminated using a solar simulator at AM 1.5 G for 10 s, for which the light intensity was adjusted to 1 sun intensity (100 mW cm^{-2}) through the use of an NREL-calibrated Si solar cell with a KG-5 filter. The J-V curves were measured along the reverse scan direction from 1.5 V to − 0.05 V or the forward scan direction from −0.05 V to 1.5 V. The step voltage and scan speed were fixed at 10 mV and 150 mV s^{-1}, respectively. The J–V curves for all devices were measured by metal shadow masks with active areas of 0.3 x 0.3 =0.09 cm^2 (small-area device) and 1×1 cm^2 (large-area device) in size.

External quantum efficiency measurement. The spectral response was taken by an spectral IPCE measurement system (K3100, McScience), which was equipped with a monochromator, a K240 XE 300 lamp source connected with K401 OLS XE300W lamp Power supply and a K102 Signal amplifier. Prior to the use of the light, the spectral response and the light intensity were calibrated using a Si-photodiode (Model: S1337-1010BQ) and InGaAs photodiode (model: G12180-050A) for 300-1100 nm and 1100-1400 nm calibration respectively. Measurements were taken in External quantum efficiency (EQE) mode.

Device stability test. All measurements were performed on un-encapsulated cells in ambient air. The device stability was tested in air at room temperature without encapsulation and after each measurement, devices were stored in ambient condition without any encapsulation. For long term stability, devices were kept in a petri dish in ambient air without any encapsulation and J-V curves were periodically measured under AM1.5 G simulated sun light at room temperature. Maximum power point tracking (MPPT) was also carried out to verify the long-term photostability. The device is held at maximum power point during aging and kept at a constant temperature by an air stream flowing onto the devices (back side), with the device surface measuring approximately 20 °C under the white LED light was measured by IVUM-state potentiostate (Ivium Technologies B.V., Eindhoven, The Netherlands). For photostability, the fabricated devices directly illuminated under 1 sun illumination in ambient conditions and recorded photocurrent which is converted into efficiency using respective bias voltage. For thermal stability, perovskite devices were kept hot-plate in ambient conditions at 60 °C and 85 °C and taken out during measurements and reported relative humidity has been mentioned each figure captions. We have not used any special maintained accessories for any environmental conditions such as room temperature or humidity, dry-air box etc. However, we monitored devices temperature ~20 °C by continuous air-steam flow, and relative humidity was ~35% for continuous illumination testing.

Table S1. Calculated hole (m^*_h) and electron (m^*_h) effective masses of $CsPbI_2Br$, $CsPb_{0.96}Eu_{0.04}I_2Br$ and $CsPb_{0.96}In_{0.04}I_2Br$ materials along high symmetry directions.

Material	Direction	m^*_h (m_e)	m^*_e (m_e)
**$CsPbI_2Br$**	Γ−R	0.0353	0.0014
	R−X	0.0022	0.0029
	X−M	0.0019	0.0029
	M−R	0.0040	0.0027
**$CsPb_{0.96}Eu_{0.04}I_2Br$**	Γ−R	0.0265	0.0017
	R−X	0.0031	0.0123
	X−M	0.0027	0.0121
	M−R	0.0022	0.0039
**$CsPb_{0.96}In_{0.04}I_2Br$**	Γ−R	0.0113	0.0025
	R−X	0.0038	0.0129
	X−M	0.0021	0.0148
	M−R	0.0068	0.0078

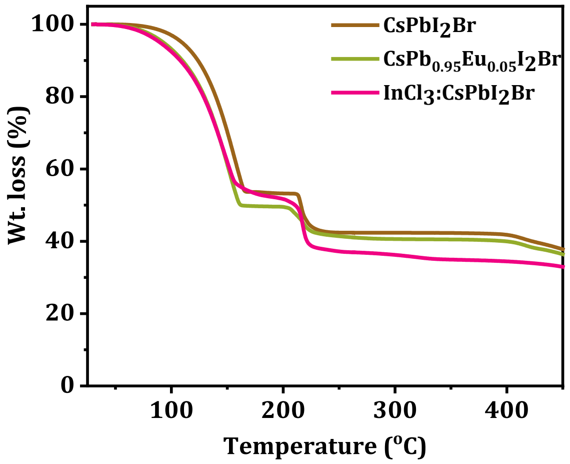

Figure S1. Thermal gravimetric analysis (TGA) of perovskite precursor in DMSO solvent.

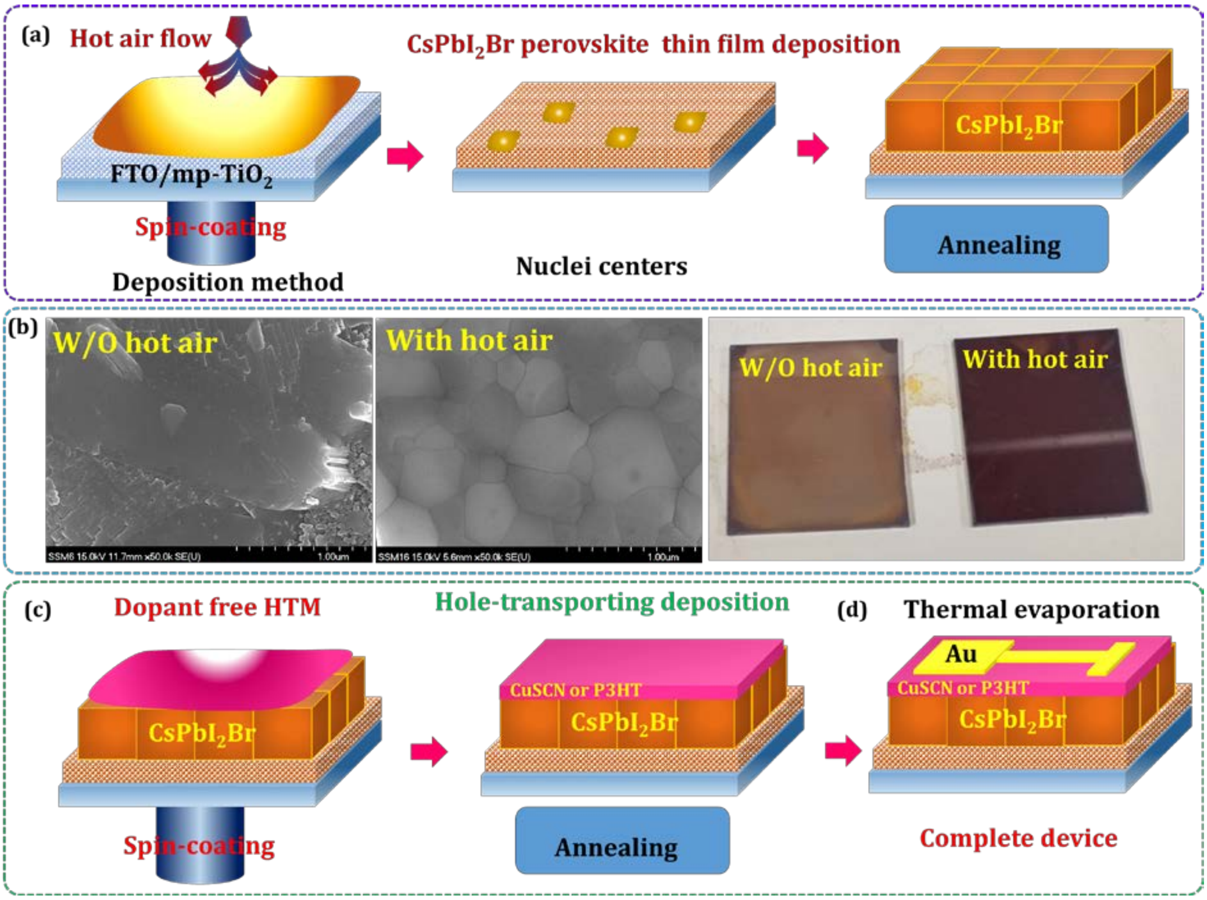

Figure S2. All-inorganic thin film deposition technique, morphology and device architecture. (a) Schematic illustration of the nucleation and crystallization procedures during the formation of perovskite films via the hot-air assisted methods. Representative SEM images of the corresponding films. (b) Optical images of the $CsPbI_2Br$ perovskite thin film fabricated by WHA and DHA method. (c) dopant-free hole-transporting material deposition and device configuration used in the present study.

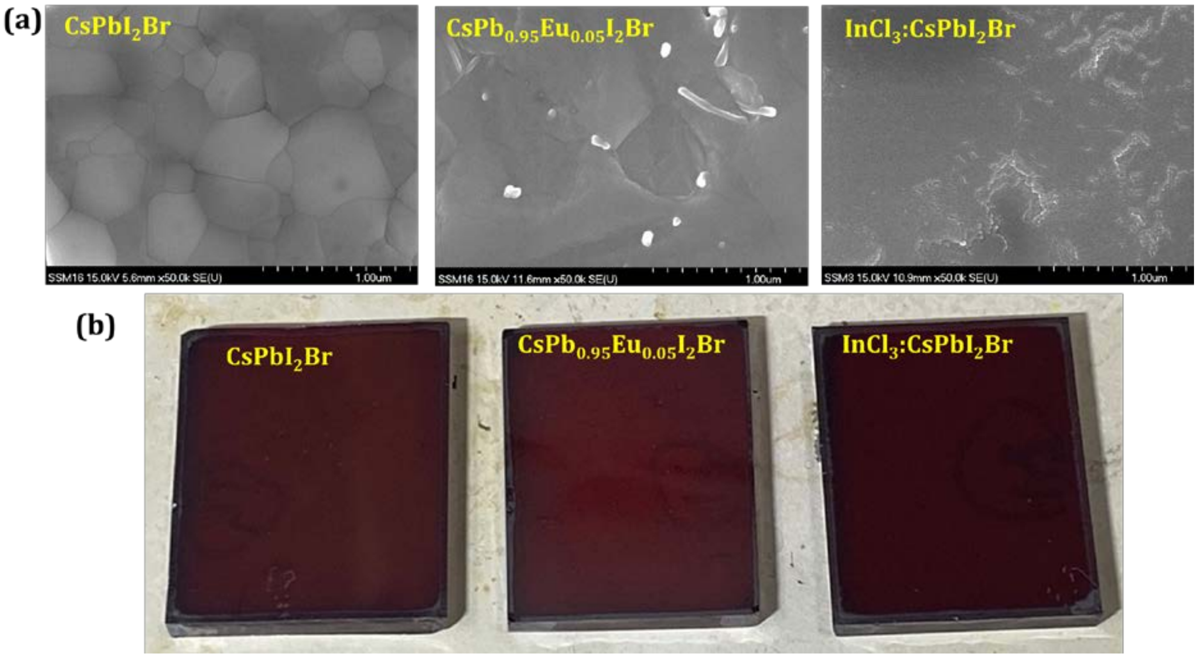

Figure S3. (a) SEM micrographs of the $CsPbI_2Br$, $CsPb_{0.95}Eu_{0.05}I_2Br$ and $InCl_3$:$CsPbI_2Br$ thin films deposited by hot-air method in ambient condition. (b) Respective photographs for each composition.

Supporting Note 1.

Initially, we have checked the thermal gravimetric analysis (TGA) of prepared perovskite precursors and optimized annealing temperature of perovskite, **Fig. S1**. Therefore, after hot-air process these samples were annealed at 280 °C for 10 min which results in crystallization of the intermediate phase into dense and pin-hole free black $CsPbI_2Br$ perovskite phase.

Fig. S2 shows a schematic illustration of the deposition of $CsPbI_2Br$-based perovskite thin film deposition by our recently developed hot-air method in ambient condition. [S9] Typically, 1.2 M perovskite precursor solution was prepared in dimethyl sulfoxide (DMSO) solvent. Here, we have chosen DMSO as a working solvent to increase the solubility of the I^- and Br^- anions and to improve the halide coordination with the solvent. The spinning conditions are 1000 and 3000 rpm at 10 and 30 s, respectively. 50 µl of the perovskite precursor solution is spread onto a fluorine doped tin-oxide (FTO) substrate coated with a compact TiO_2/mesoporous TiO_2 layer (FTO/c-TiO_2/mp-TiO_2) (1.5 cm × 2.5 cm). 1 cm distance perpendicular to the substrate was maintained between hot-air gun head and substrate surface, **Fig. S2a**. A hot-air gun is used during the spin coating process to partially remove the DMSO

solvent while the substrate is spinning and to favor the formation of the solid PbX_2-DMSO-CsI intermediate phase. Hot-air gun temperature was 150 °C up to light-brown film formation, which was adopted by our previous optimized conditions. [S9, S10] The use of DMSO led to the formation of the intermediate PbX_2-DMSO-CsI complex phase which limits the initial number of nuclei centers being controlled by the specific solvent evaporation rate.

Fig. S2b exhibits the SEM images of the $CsPbI_2Br$ films deposited on an electron transport layer (ETL) substrate with and without hot-air (150 °C). It can be observed that in the films deposited with the W/O hot-air method, the $CsPbI_2Br$ perovskite film was not covered adequately and the crystals are randomly distributed due to the fast DMSO evaporation. However, the hot-air method yielded homogeneous $CsPbI_2Br$ perovskite grains covered completely the mp-TiO_2 layer. The photographs of the processed films prepared with the hot air method are clear more intense color which indicates highly uniform nature.

Top-view SEM images in **Fig. S3a** apparently exhibit the formation of highly uniform, pinhole free $CsPb_{0.95}Eu_{0.05}I_2Br$ and $InCl_3$:$CsPbI_2Br$ thin films. This is also reflected from the photographs of respective samples, **Fig. S3b.** This highly uniform film surface would be favorable for smooth perovskite/HTL interfaces which reduces the carrier recombination. The grain size was reduced after Eu and In doping which is usually observed for metal cation doped $CsPbI_2Br$ samples.

Annealed samples were further used for dopant-free CuSCN or P3HT deposition. For CuSCN deposition, 35 $mgml^{-1}$ in diethyl sulfide (DES) was spin coated dynamically on to $CsPbI_2Br$ film at 5000 rpm for 30 s and annealed at 100 °C for 10 min in order to remove DES residue. In order to protect the perovskite layer from gold diffusion, an rGO interfacial layer was applied after CuSCN HTL. In case of dopant-free P3HT HTL, 15 mg of P3HT dissolved in 1 ml chlorobenzene (CB) solvent and spin coated at 3000 rpm for 30 s. P3HT deposited samples were further annealed at 200 °C for 5 min in order to remove the CB solvent and crystalize the P3HT. This annealing process not only remove the CB solvent but also reduce the energy disorder in P3HT film and improve the hole-injection properties.

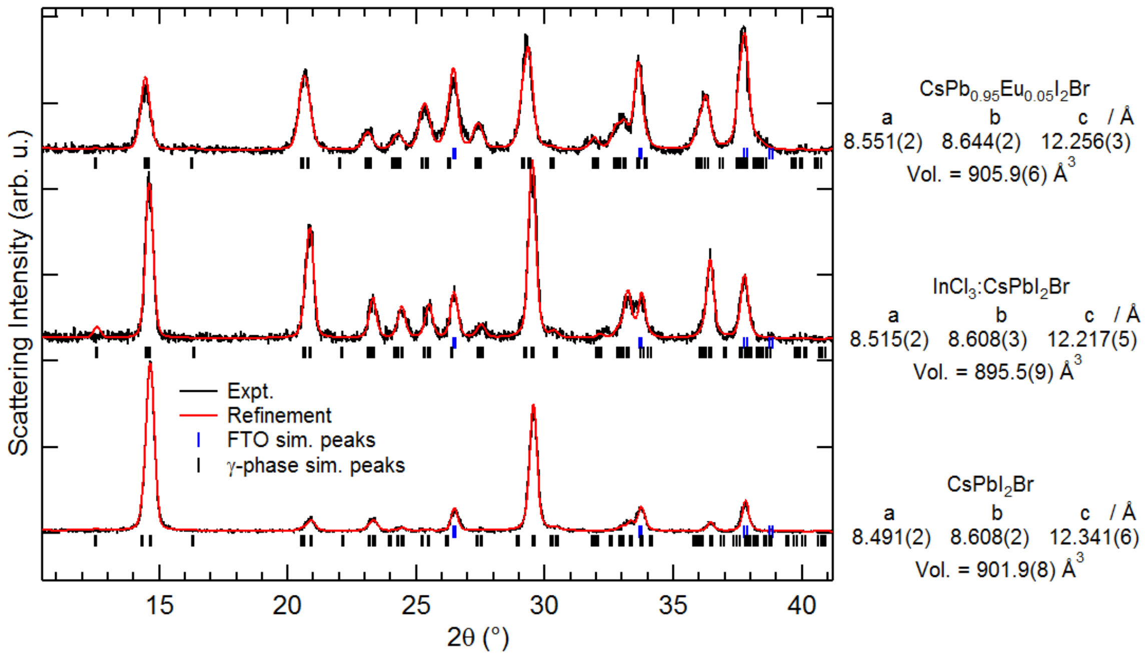

Figure S4: Normalized XRD patterns and their structural refinements (La Bail method) of γ-phase $CsPbI_2Br$-based thin films with the derived lattice parameters presented on the right. The numbers in parentheses represent the uncertainty in units of the last significant figure. For clarity, the scattering data have been background corrected and offset.

Supporting Note 2:

Refinement of XRD data of the black phase indicate a distorted orthorhombic γ-phase perovskite structure (*Pbmn*) with lattice constants a=8.491(2), b=8.608 (2) and c=12.341 (6) Å with lattice volume 901.1 (8) $Å^3$. After 0.25 % In^{3+} and Cl^- doping we found a slight decrease in the lattice volume 895.5 (9) $Å^3$, yielding appears a=8.515 (2), b=8.615 (3) and c=12.217 (9) Å. This smaller volume arises due to the substitution of Pb^{2+} (1.19 Å) by In^{3+} (0.91 Å) and Br^- (1.96 Å) by Cl^- (1.81 Å). [S11] On the other hand, we observed small increment in the lattice volume up to 905.9 (6) $Å^3$, with lattice constants: a=8.551 (2), b=8.644 (2) and c=12.256 (3) Å.

Table S2. PLQY values for $CsPbI_2Br$ and $CsPb_{0.95}Eu_{0.05}I_2Br$ thin films deposited on Glass, P3HT, CuSCN and c-TiO_2/mp-TiO_2 substrate.

**Substrate**	**$PLQY_{CsPbI_2Br}$ (%)**	**$\Delta QFLS_{rad}$ pristine (meV)**	**$PLQY_{Eu}$ (%)**	**$\Delta QFLS_{rad}$ Eu (meV)**	**$\Delta QFLS_{Eu}$ (meV)**
Glass	0.51	135	0.91	121	14.88
P3HT	0.26	153	0.53	135	18.30
CuSCN	0.28	151	0.48	137	13.85
TiO_2	0.12	173	0.28	151	21.78

Table S3. PLQY values for $CsPbI_2Br$ and $InCl_3$:$CsPbI_2Br$ thin films deposited on Glass, P3HT, CuSCN and c-TiO_2/mp-TiO_2 substrate.

**Substrate**	**PLQY $_{CsPbI_2Br}$ (%)**	**$\Delta QFLS_{rad}$ pristine (meV)**	**$PLQY_{InCl3}$ (%)**	**$\Delta QFLS_{rad}$ $InCl_3$ (meV)**	**$\Delta QFLS_{InCl_3}$ (meV)**
Glass	0.51	135	0.87	122	13.73
P3HT	0.26	153	0.60	131	21.49
CuSCN	0.28	151	0.56	133	17.81
TiO_2	0.12	173	0.29	150	22.68

Supporting Note 3:

For a standard PV material, the change in QFLS following passivation ($\Delta QFLS = QFLS_{doped} - QFLS_{controlled}$) is given by:[S12, S13]

$$\Delta QFLS = 25.7meV \times \ln\left(\frac{PLQY_{doped}}{PLQY_{controlled}}\right) \quad \text{..... (S1)}$$

From equation S1 we can easily calculate the expected increase in open-circuit voltage for a given PLQY ratio.

The above equation S1 can be modified to calculate the bandgap-independent loss from the radiative limit. By setting $PLQY_{doped}$ sample to be unity, i.e., in the ideal case, and then inputting the measured PLQY ($PLQY_{absolute}$), we obtain

$$\Delta QFLS_{rad} = 25.7 meV \times \ln\left(\frac{1}{PLQY_{absolute}}\right) \quad \text{.... (S2)}$$

where $PLQY_{absolute}$ is expressed in measured absolute value.

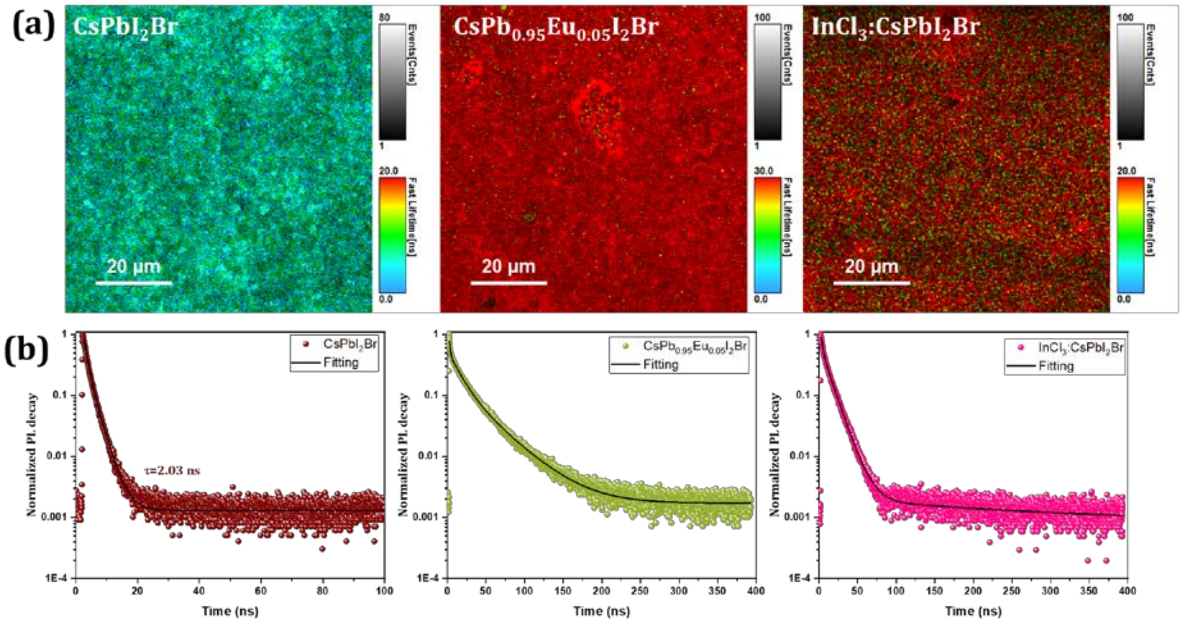

Figure S5. (a) Fluorescence-lifetime imaging microscopic (FLIM) images (80 µm x 80 µm, λ_{ex}=470 nm) of the $CsPbI_2Br$, $CsPb_{0.95}Eu_{0.05}I_2Br$ and $InCl_3$:$CsPbI_2Br$, thin films. The FLIM images were analyzed using a three-exponential decay model: τ_{avg}, images constructed on the basis of the averaged photoluminescence lifetimes; τ_1, τ_2, and τ_3 indicate images constructed on the basis of the fast, mid-range, and slow-components, respectively; $\tau_1 + \tau_2 + \tau_3$, overlay images. (b) TRPL decay profiles.

Supporting Note 4:

The time-resolved photoluminescence (TRPL) decay profile data was fitted to a tri-exponential function of the form:

$$I(t) = I_0 + A_1 \exp\left(-\frac{t-t_0}{\tau_1}\right) + A_2 \exp\left(\frac{-t-t_0}{\tau_2}\right) + A_3 \exp\left(\frac{-t-t_0}{\tau_3}\right) \quad \text{(S3)}$$

where, τ_1, τ_2 and τ_3 are first, second and third order decay time, A_1, A_2 and A_3 are respective weight factors of each decay channel. The average recombination lifetimes $< \tau_{avg} >$ for

respective samples were calculated from lifetime values and weight fraction amplitude values (%) using the following equation:

$$< \tau_{avg} >= \sum_{n} \frac{\Sigma A_n \tau_n^2}{\Sigma_m A_m \tau_m^2} \quad \text{(S4)}$$

The non-radiative fast-decay lifetime (τ_1 and τ_2) and radiative slow-decay lifetime (t_3) originated from the quenching of charge carriers and free charge carriers before the charge collection, respectively.

Table S4. TRPL lifetime measurements of inorganic perovskite absorber layers deposited on FTO/c-TiO_2/mp-TiO_2 ETL substrates. Weight fraction calculated from amplitude at particular lifetime decay.

**Perovskite composition**	**t_1 (ns)**	**t_2 (ns)**	**t_3 (ns)**	**A_1***	**A_2***	**A_3***	**<t> (ns)**
$CsPbI_2Br$	1.08	2.67	-	3.1	5.5	-	2.03
$CsPb_{0.95}Eu_{0.05}I_2Br$	1	14	34	3.3	3.17	1.5	27
0.25 % $InCl_3$:$CsPbI_2Br$	2.21	11.8	172	5.59	3.86	0.014	16

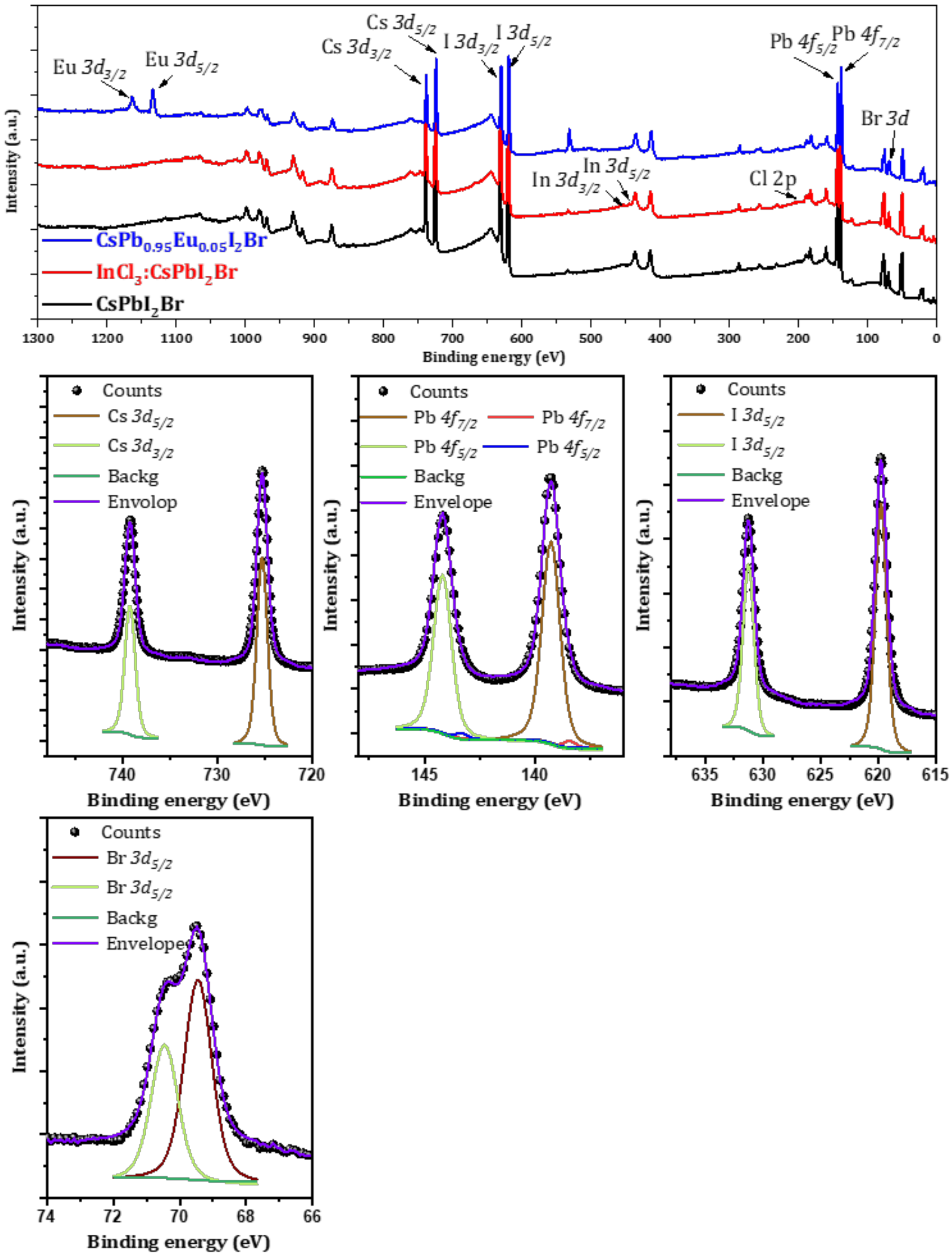

Figure S6. Survey spectra and XPS fittings for the Cs *3d*, Pb *4f*, I *3d* and Br *3d* core levels for the controlled $CsPbI_2Br$ sample. The counts and envelope have been offset to make the fittings clearer. Full details of peak positions can be found in **Table S5**.

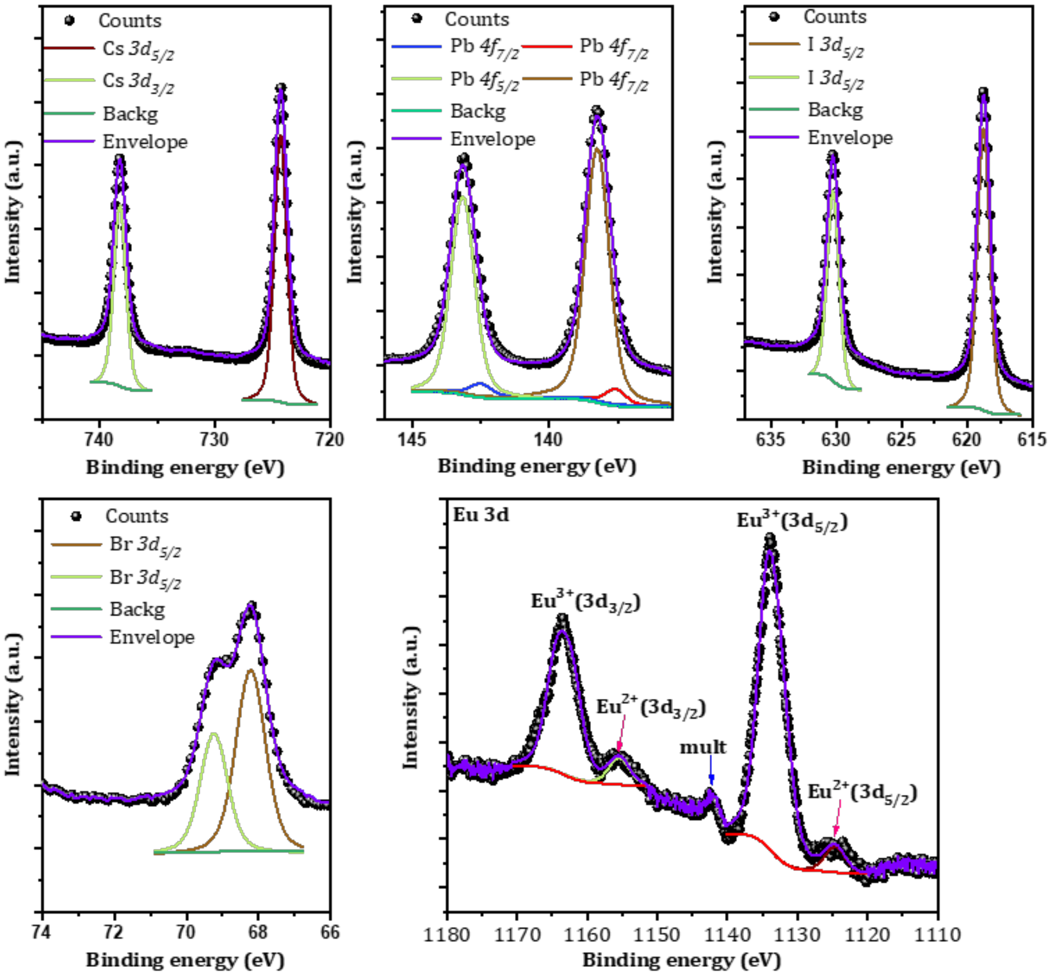

Figure S7. XPS fittings for the Cs *3d*, Pb *4f*, I *3d*, Br *3d* and Eu *3d* core levels for the controlled $CsPb_{0.95}Eu_{0.05}I_2Br$ sample. The counts and envelope have been offset to make the fittings clearer. Full details of peak positions can be found in **Table S5**.

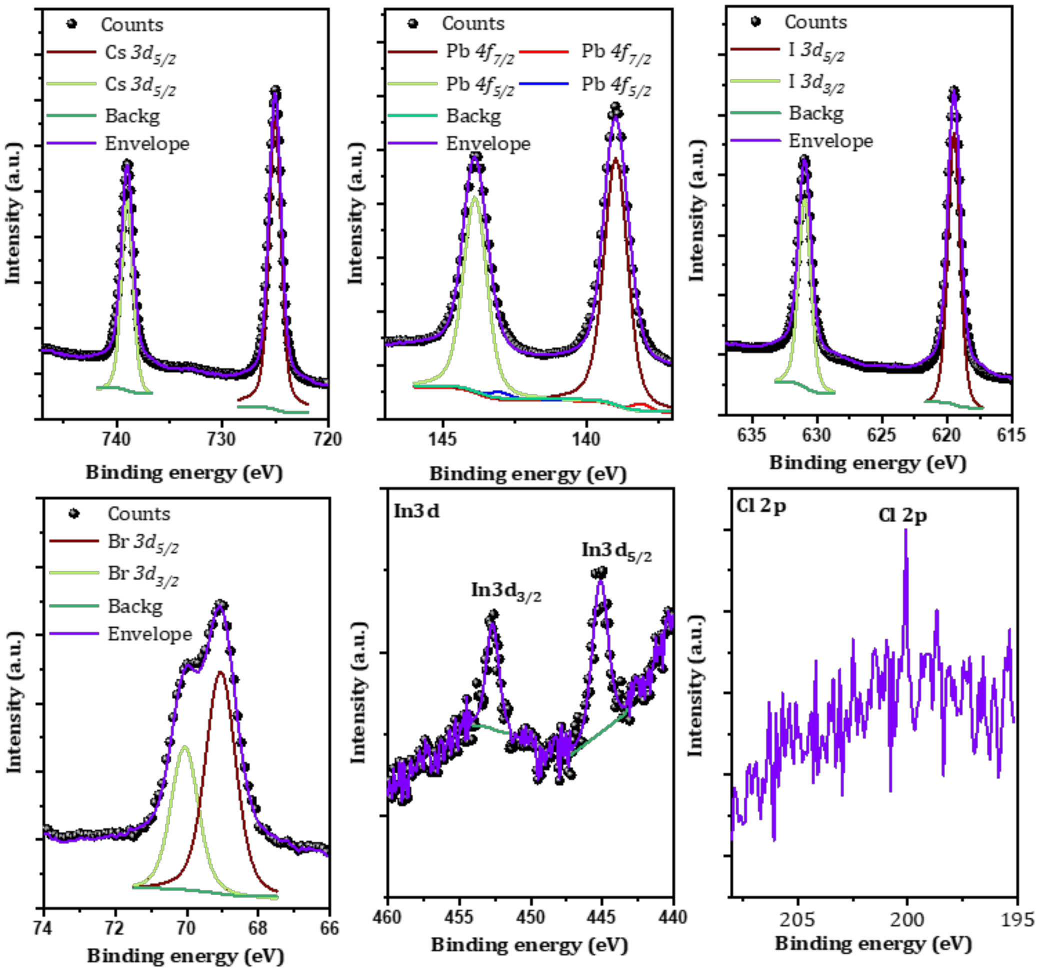

Figure S8. XPS fittings for the Cs *3d*, Pb *4f*, I *3d*, Br *3d*, In *3d* and Cl *2p* core levels for the controlled $InCl_3$:$CsPbI_2Br$ sample. The counts and envelope have been offset to make the fittings clearer. Full details of peak positions can be found in **Table S5**.

Supporting Note 5:

The analysis of Eu peaks is little tricky due to its Eu^{2+} and Eu^{3+} oxidation states. We observed two prominent peaks in both groups denoted by trivalent Eu^{3+} and divalent Eu^{2+} and a small structure in the $3d_{5/2}$ region denoted by ''*mult*''. This additional peak assignment was done by Cho *et al*. based on their inelastic energy loss and surface and bulk plasmon. [S14] Furthermore, the lower BE peak-shift in I *3d* and Br *3d* core-levels confirms the Eu^{3+}-Eu^{2+} ion pair working as a redox shuttle which terminates the degradation. [S15] The presence of the Eu^{3+} element is reflected from the doublet peak appears at 1133.92 and 1163.6 eV respectively for Eu^{3+} $3d_{5/2}$ and Eu^{3+} $3d_{3/2}$ core levels. While divalent Eu^{2+} is revealed from doublet peaks at Eu^{2+} $3d_{5/2}$ (1124.62 eV) and Eu^{2+} $3d_{3/2}$ (1155.45 eV) core levels, **Fig. 2d**.

Table S5. XPS core-level peak positions of $CsPbI_2Br$, $CsPb_{0.95}Eu_{0.05}I_2Br$ and 0.25 % $InCl_3$:$CsPbI_2Br$ thin films.

**Perovskites**	**$CsPbI_2Br$**			**$CsPb_{0.95}Eu_{0.05}I_2Br$**			**0.25 % $InCl_3$:$CsPbI_2Br$**		
Core levels	Start BE (eV)	Peak BE (eV)	End BE (eV)	Start BE (eV)	Peak BE (eV)	End BE (eV)	Start BE (eV)	Peak BE (eV)	End BE (eV)
**Cs $3d_{5/2}$**	728.28	725.31	722.68	727.68	724.33	721.18	728.48	725.05	721.98
**Cs $3d_{3/2}$**	742.08	739.24	736.38	740.78	738.27	735.58	741.78	738.99	736.68
**Pb $4f_{7/2}$**	146.28	139.29	136.98	145.18	138.27	135.58	145.98	138.96	136.08
**Pb $4f_{5/2}$**	146.28	144.19	136.98	145.18	146.17	135.58	145.98	143.86	136.08
**I3 $d_{5/2}$**	622.38	619.78	617.18	621.58	618.76	616.18	621.68	619.48	617.28
**I3 $d_{3/2}$**	633.48	631.26	629.08	632.48	630.24	628.08	633.11	630.96	628.68
**Br $3d_{5/2}$**	71.98	69.45	67.68	70.68	68.2	66.48	71.48	70.06	67.48
**Br $3d_{3/2}$**	71.98	70.46	67.68	70.68	69.22	66.48	71.48	69.05	67.48
**$Eu^{2+}3d_{5/2}$**	-	-	-	1140.18	1124.62	1120.08	-	-	-
**Eu^{2+} $3d_{3/2}$**	-	-	-	1170.68	1155.45	1151.58	-	-	-
**Eu^{3+} $3d_{5/2}$**				1140.18	1133.92	1120.08			
**Eu^{3+} $3d_{3/2}$**				1170.68	1163.6	1151.58			
mult					1142.09				
**In $3d_{5/2}$**	-	-	-	-	-	-	447.18	445.16	443.28
**In $3d_{3/2}$**	-	-	-	-	-	-	454.08	452.67	451.48
**Cl *2p2***	-	-	-	-	-	-	205	200.79	197.23

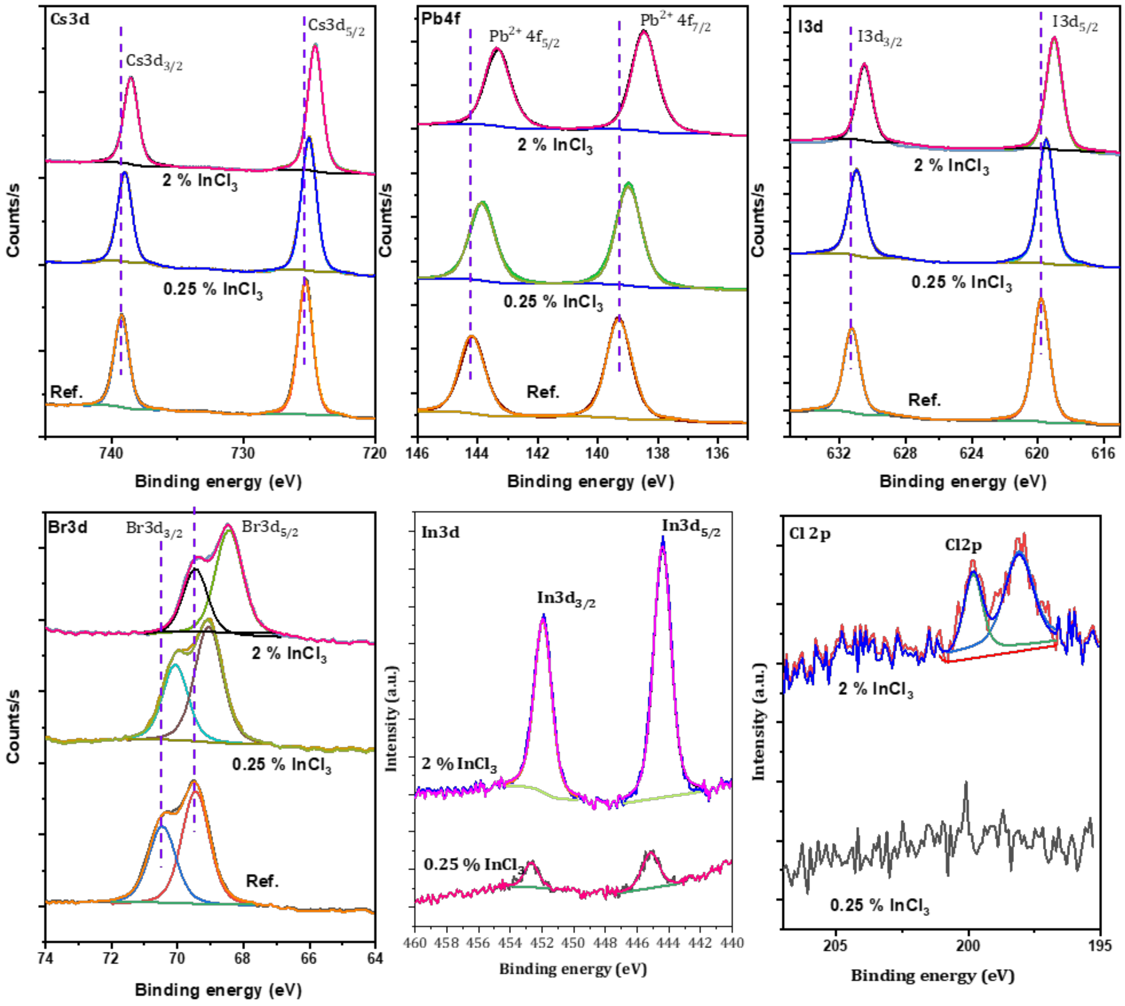

Figure S9. XPS fittings for the Cs *3d*, Pb *4f*, I *3d*, Br *3d*, In *3d* and Cl *2p* core levels for the controlled $InCl_3$:$CsPbI_2Br$ sample. The counts and envelope have been offset to make the fittings clearer. Full details of peak positions can be found in **Table S6**.

Table S6. XPS core-level peak positions of $CsPbI_2Br$, 0.25 % $InCl_3$:$CsPbI_2Br$ and 2 % $InCl_3$:$CsPbI_2Br$ thin films.

**Perovskites**	**$CsPbI_2Br$**			**0.25 % $InCl_3$:$CsPbI_2Br$**			**2 % $InCl_3$:$CsPbI_2Br$**		
Core levels	Start BE (eV)	Peak BE (eV)	End BE (eV)	Start BE (eV)	Peak BE (eV)	End BE (eV)	Start BE (eV)	Peak BE (eV)	End BE (eV)
**$Cs3d_{5/2}$**	728.28	725.31	722.68	728.48	725.05	721.98	729	724.27	719
**$Cs3d_{3/2}$**	742.08	739.24	736.38	741.78	738.99	736.68	741.67	738.21	734.53
**$Pb4f_{7/2}$**	146.28	139.29	136.98	145.98	138.96	136.08	140.65	138.14	135.3
**$Pb4f_{5/2}$**	146.28	144.19	136.98	145.98	143.86	136.08	146.75	143.14	140.65
**$I3d_{5/2}$**	622.38	619.78	617.18	621.68	619.48	617.28	622.93	618.96	613.44
**$I3d_{3/2}$**	633.48	631.26	629.08	633.11	630.96	628.68	634.17	630.21	626.23
**$Br3d_{5/2}$**	71.98	69.45	67.68	71.48	70.06	67.48	70.98	68.44	66.88
**$Br3d_{3/2}$**	71.98	70.46	67.68	71.48	69.05	67.48	70.98	69.46	66.88
**$In3d_{5/2}$**	-	-	-	447.18	445.16	443.28	446.88	444.41	442.08
**$In3d_{3/2}$**	-	-	-	454.08	452.67	451.48	454.28	451.94	449.78
**Cl2p2**	-	-	-	205	200.79	197.23	201.08	199.82	196.68

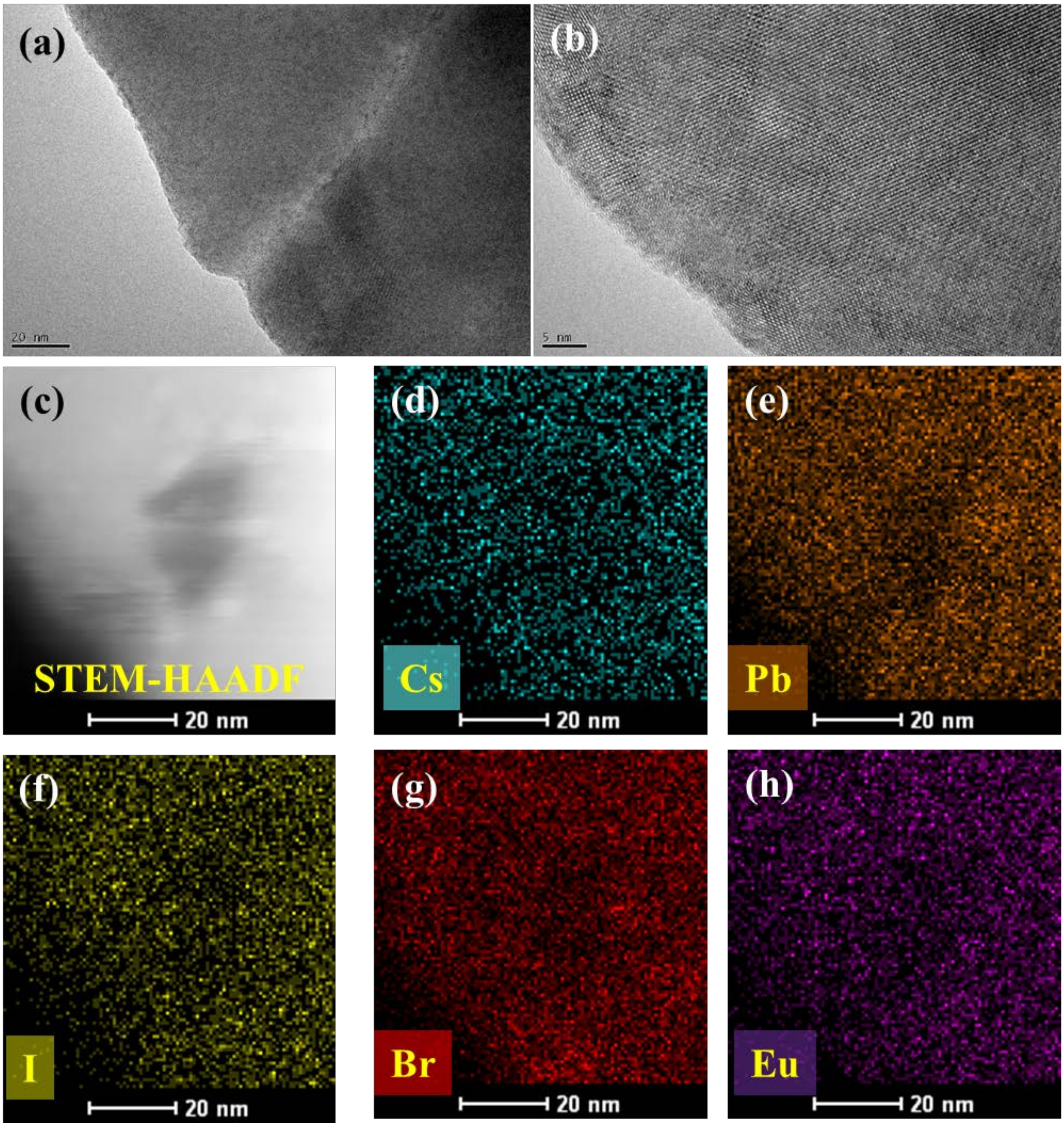

Figure S10. Structural and elemental analysis of $CsPb_{0.95}Eu_{0.05}I_2Br$ perovskite thin film (a,b) typical TEM images of the $CsPb_{0.95}Eu_{0.05}I_2Br$ perovskite sample at different magnification. (c) Respective HAADF STEM image and (d-h) elemental distribution of $CsPb_{0.95}Eu_{0.05}I_2Br$. Cyan: cesium; orange: lead; yellow: iodine; red: bromide; violet: europium. Scale bars, 20 nm.

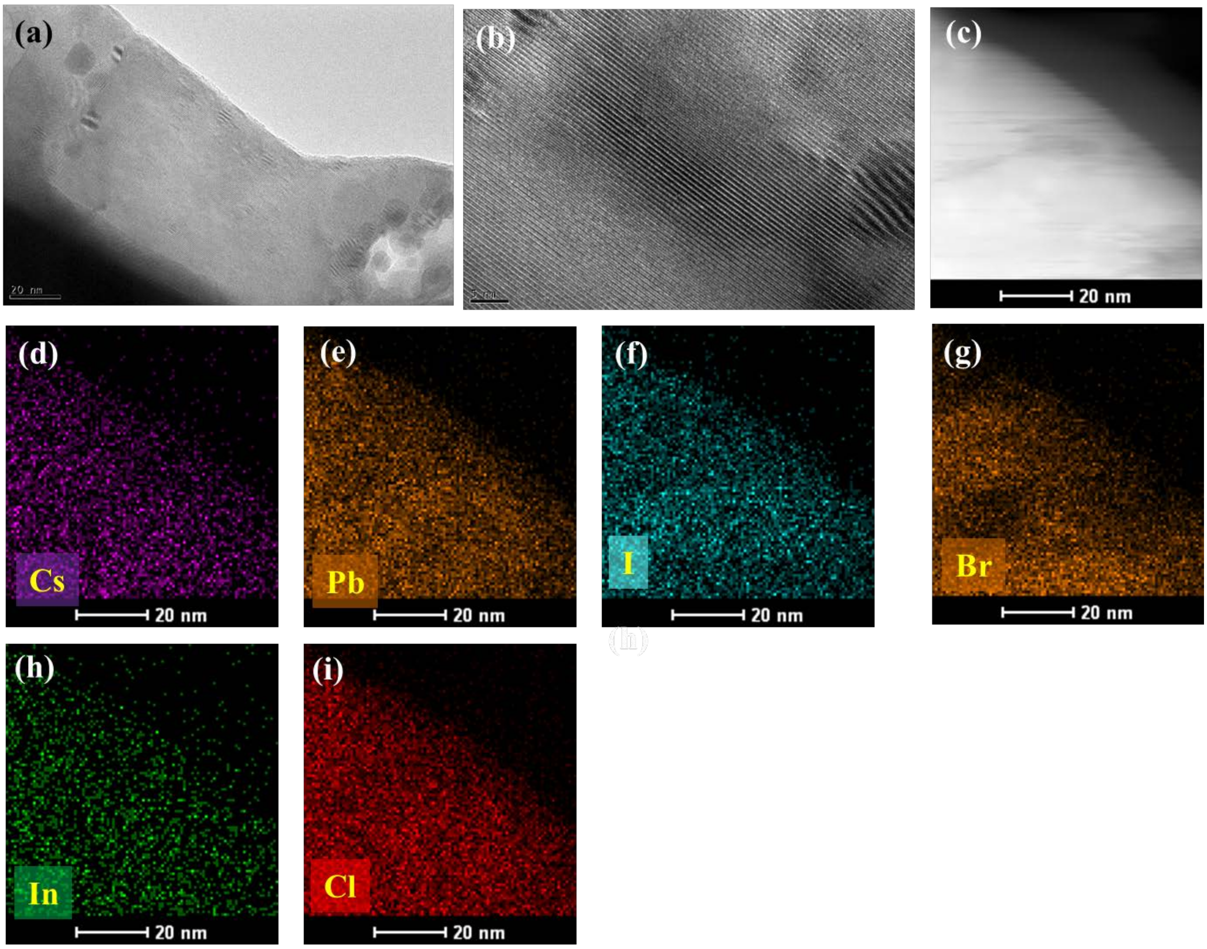

Figure S11. Structural and elemental analysis of $InCl_3$:$CsPbI_2Br$ perovskite thin film (a, b) typical TEM images of the 0.25 % $InCl_3$:$CsPbI_2Br$ perovskite sample at different magnifications (c) respective HAADF STEM image and (d-i) elemental distribution of $InCl_3$:$CsPbI_2Br$. Violet: cesium; orange: lead; cyan: iodine; yellow: bromide; light green: indium; red: chlorine. Scale bars, 20 nm.

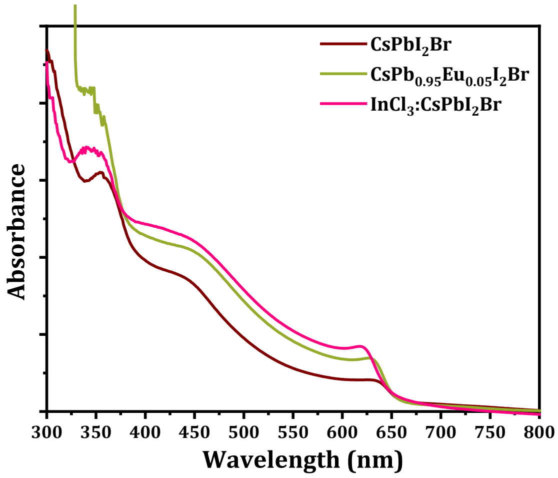

Figure S12. UV-Vis optical absorption spectra of $CsPbI_2Br$, $CsPb_{0.95}Eu_{0.05}I_2Br$ and 0.25% $InCl_3$:$CsPbI_2Br$ thin films.

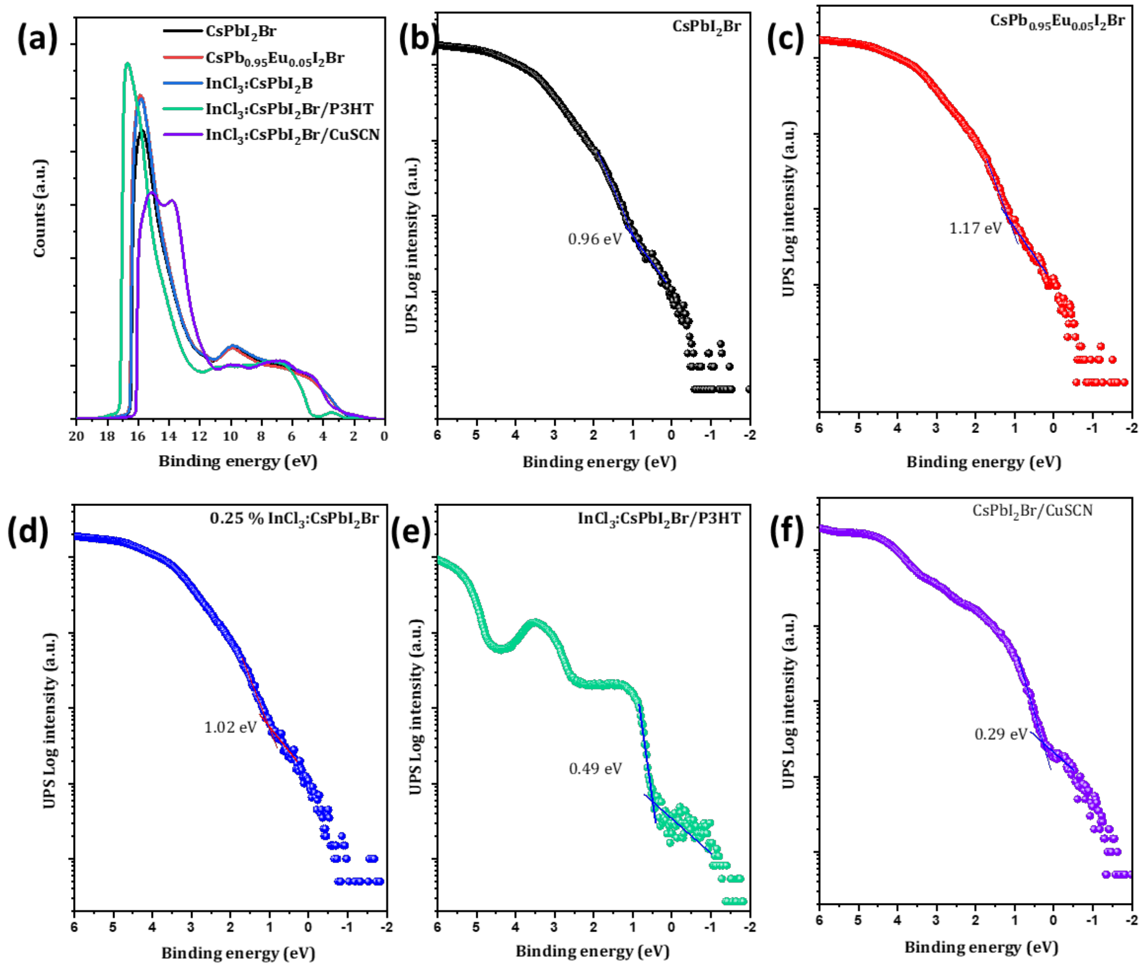

Figure S13. (a) UPS survey spectra and (b-f) valence band region of the $CsPbI_2Br$, $CsPb_{0.95}Eu_{0.05}I_2Br$, 0.25 % $InCl_3$:$CsPbI_2Br$, 0.25 % $InCl_3$:$CsPbI_2Br$/P3HT, 0.25 % $InCl_3$:$CsPbI_2Br$/CuSCN on FTO/c-TiO_2/mp-TiO_2.

Table S7. Calculated parameters for energy level of $CsPbI_2Br$, $CsPb_{0.95}Eu_{0.05}I_2Br$, 0.25 % $InCl_3$:$CsPbI_2Br$, $InCl_3$:$CsPbI_2Br$/P3HT and $InCl_3$:$CsPbI_2Br$/CuSCN from UPS analysis.

**Sample**	**E_{cutoff} (eV)**	**WF (ϕ) (eV)**	**E_F (eV)**	**VBM (eV)**	**ΔE_g (eV)**	**CB (eV)**
TiO_2	17.56	3.66	3.43	7.09	3.20	3.89*
$CsPbI_2Br$	16.50	4.72	0.96	5.68	1.913	3.767
$CsPb_{0.95}Eu_{0.05}I_2Br$	16.54	4.68	1.17	5.85	1.925	3.925
$InCl_3$:$CsPbI_2Br$	16.52	4.70	1.02	5.72	1.914	3.806
$InCl_3$:$CsPbI_2Br$/P3HT	17.19	4.03	0.49	4.52	1.94	2.58
$CsPbI_2Br$/CuSCN	16.14	5.08	0.29	5.37	3.92	1.45

**Note: As per previous literature [Ref. S9]*

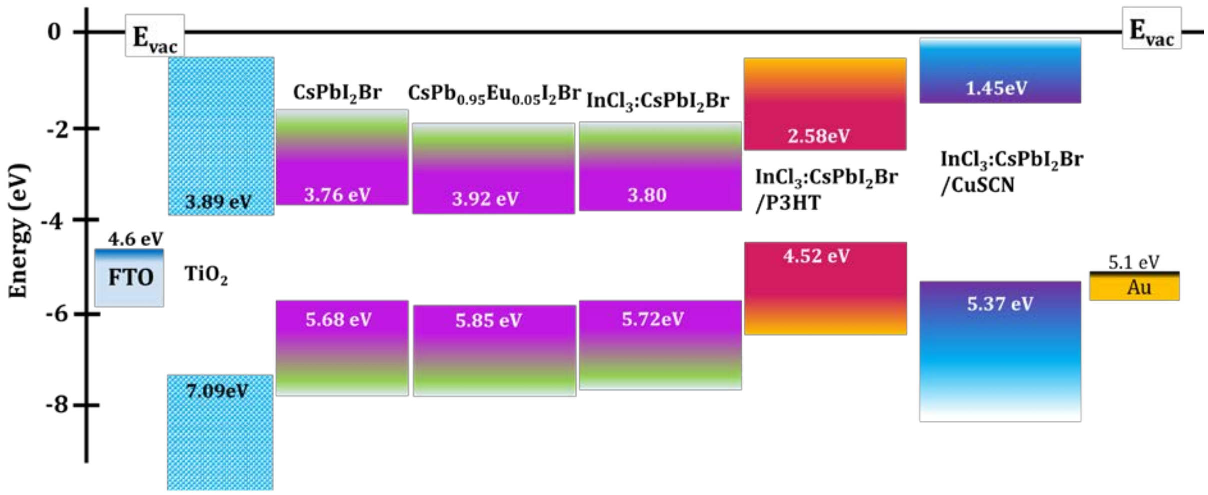

Figure S14. Energy level diagram of each layer of the perovskite device used for fabrication calculated from UPS analysis.

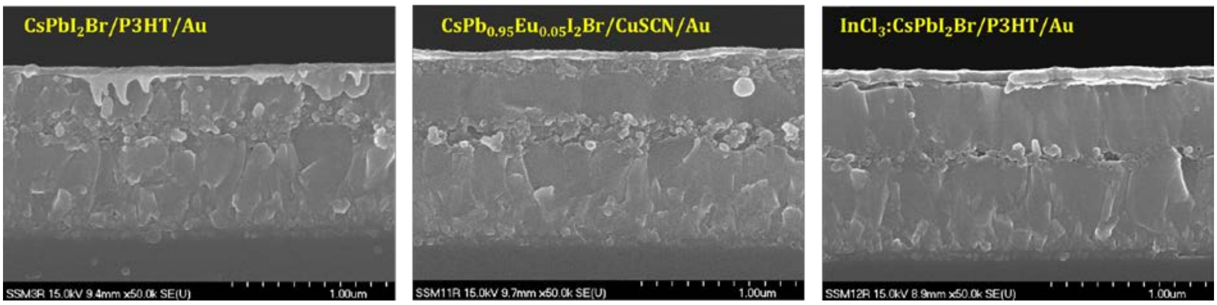

Figure S15. Cross-sectional SEM micrographs of the $CsPbI_2Br$, $CsPb_{0.95}Eu_{0.05}I_2Br$ and $InCl_3$:$CsPbI_2Br$-based PSCs with different HTLs.

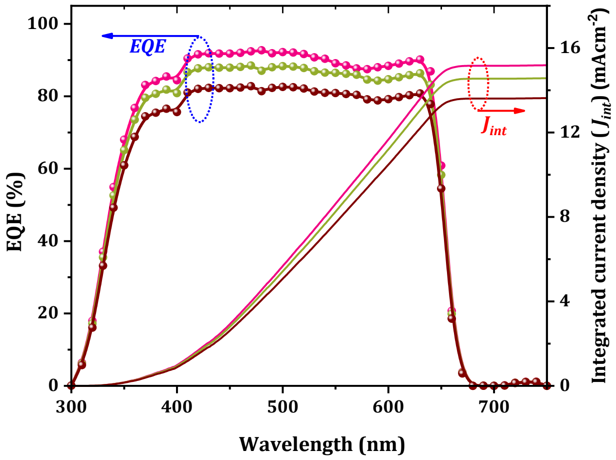

Figure S16. EQE spectra of doped $CsPbI_2Br$ perovskite thin films based on CuSCN dopant-free HTM.

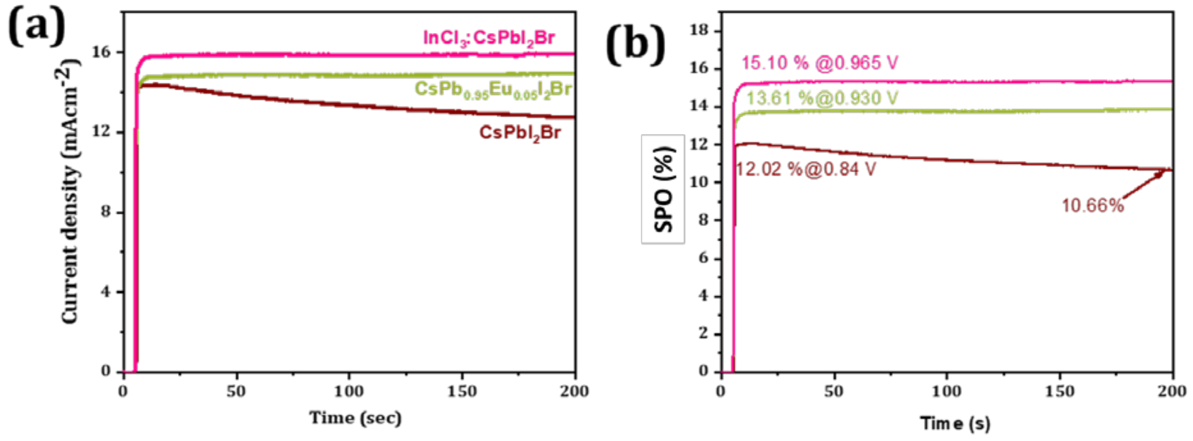

Figure S17. (a) Steady-state current density measured for 200 s at a fixed voltage maximum (V_{max}) current identified in the J–V curves and (b) stabilized power output (SPO).

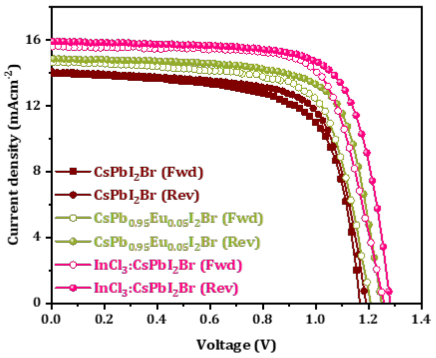

Figure S18. Hysteresis analysis of $CsPbI_2Br$, $CsPb_{0.95}Eu_{0.05}I_2Br$ and 0.25 % $InCl_3$: $CsPbI_2Br$-based perovskite solar cells using CuSCN HTM.

Table S8. Solar cell parameters of $CsPbI_2Br$, $CsPb_{0.95}Eu_{0.05}I_2Br$ and 0.25 % $InCl_3$: $CsPbI_2Br$-based perovskite solar cells using CuSCN HTM recorded under forward and reverse scan mode.

Sample	Scan direction	V_{OC} (V)	J_{SC} (mAcm^{-2})	FF (%)	PCE (%)
$CsPbI_2Br$	Forward	1.166	13.95	68.20	11.09
	Reverse	1.192	14.06	71.68	12.01
$CsPb_{0.95}Eu_{0.05}I_2Br$	Forward	1.207	14.63	70.88	12.51
	Reverse	1.249	14.90	73.76	13.72
0.25 % $InCl_3$:$CsPbI_2Br$	Forward	1.262	15.61	72.42	14.26
	Reverse	1.282	15.91	74.85	15.27

Table S9. Hysteresis analysis of inorganic perovskite solar cells based on dopant free P3HT HTMs.

**Sample**	**HTM**	**Scan**	**V_{OC} (V)**	**J_{SC} (mAcm^{-2})**	**FF (%)**	**PCE (%)**
0.25 % $InCl_3$:$CsPbI_2Br$	P3HT	Forward	1.277	15.90	73.10	14.83
		Reverse	1.303	15.90	75.76	15.69

KITECH
Korea Institute of Industrial Technology
89, Yangdaegiro-gil, Ipjang-myeon, Seobuk-gu, Cheonan-si,
Chungcheongnam-do 31056 Republic of Korea
Tel: +82-41-589-8114 | Fax: +82-41-589-8120
www.kitech.re.kr

TEST REPORT

1. Certificate No.	:	C20N300077-01-01-(00-00)
2. Client	:	Chonnam National University, Polymer Energy materials laboratory<br>77, Yongbong-ro, Buk-gu, Gwangju, Republic of Korea
3. Date of Test	:	Jan.07.2021 ~ Jan.07.2021
4. Usage	:	For paper Submission
5. Sample Description	:	Inorganic Perovskite Solar Cells
		* The results shown in this report refer only to the sample(s) tested unless otherwise stated.
6. Test Method Used	:	KS C IEC 60904-1
7. Test Result	:	Refer to the test results
8. Test Site	:	Korea Institute of Industrial Technology, Seonam Regional Division, 6, Cheomdangwagi-ro 208 beon-gil, Buk-gu, Gwangju, Republic of Korea

Affirmation	Tested by<br>Name : Jeong Hyejeong (signature)<br>E-mail : jeong124@kitech.re.kr	Technical Manager<br>Name : BOO SEONG JAE (signature)<br>E-mail : sboo@kitech.re.kr

Jan.13.2021

PRESIDENT of KOREA INSTITUTE OF INDUSTRIAL TECHNOLOGY

1. The results recorded in this test report is limited to the test results produced from the sample submitted and the test report does not guarantee the quality of overall samples.
2. This test report shall not be used for PR, advertisement, lawsuit and any other purpose outside the scope of its defined usage.
3. The authenticity of this test report can be checked QR code.

KITECH
Korea Institute of Industrial Technology
89, Yangdaegiro-gil, Ipjang-myeon, Seobuk-gu, Cheonan-si,
Chungcheongnam-do 31056 Republic of Korea
Tel: +82-41-589-8114 | Fax: +82-41-589-8120
www.kitech.re.kr

Test Result

1. Test Sample
   ◦ Sample name : Inorganic Perovskite Solar Cells

2. Test condition
   ◦ Equipment : Solar simulator (Wacom, WXS-155S-L2)
   ◦ Temperature : (23 ± 1) ℃, Relative Humidity : (25 ± 1) % R.H.

3. Test Results ※

Test Items	Unit	Test Result	Remarks
maximum power (Pmax)	mW	1.35	
short-circuit current (Isc)	mA	1.44	
open-circuit voltage (Voc)	V	1.24	
fill factor (FF)	%	75.64	
maximum power voltage (Vpm)	V	1.03	
maximum power current (Ipm)	mA	1.30	
cell efficiency (Eff)	%	14.97	
cell area	㎠	0.09	

※ 25 ℃, 100 mW/㎠

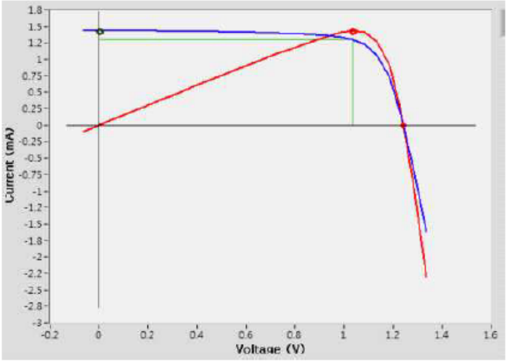

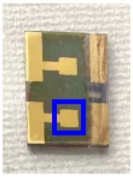

Sample image

[QP-17-04B]
C20N300077-01-01-(00-00)

Figure S19. The certified result of dopant free P3HT based device having $InCl_3$:$CsPbI_2Br$ composition based perovskite solar cell measured using Solar simulator (Wacom, WXS-155S-L2), Measurement temperature was (23 ± 1) ℃ with Relative Humidity (25 ± 1) % at Korea Institute of Industrial Technology (KITECH). The device exhibits a certified PCE of 14.97 % with V_{OC} of 1.24 V, I_{SC} of 1.44 mA cm^{-2}, FF of 75.64 %. In order to avoid over estimation of current density identical thin SS-metal mask (0.09 cm^2) was attached from the backside of the device. The J-V curve was recorded in reverse scan from 1.4 V to -0.2 V, the voltage step: 0.05 V, Compliance: 10 mA, Source Delay: 0 sec and Scan Speed: Normal speed.

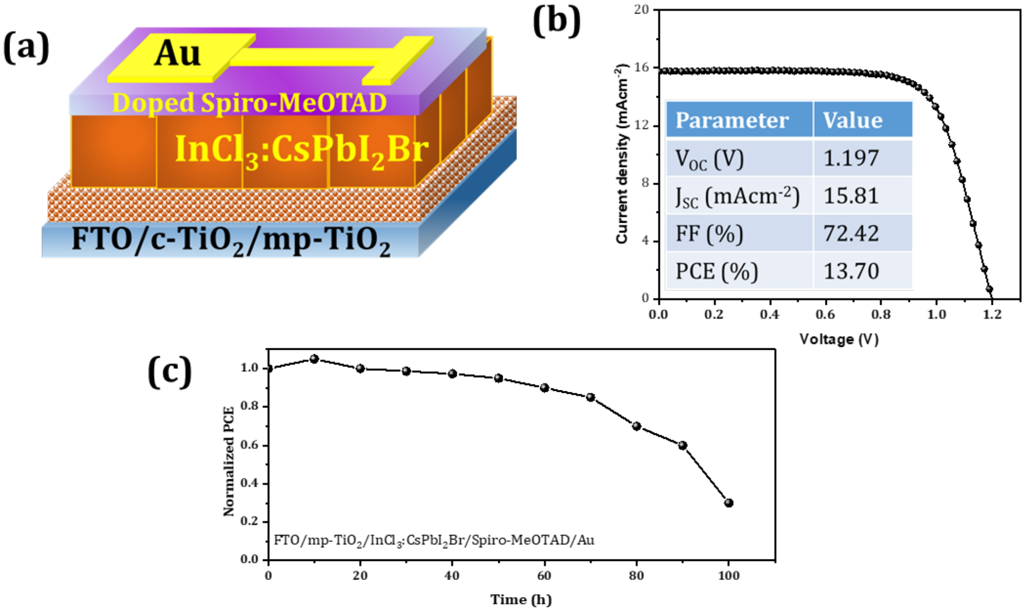

Figure S20. Conventional Spiro-MeOTAD based inorganic solar cells and its stability in ambient conditions (a) device architecture used in the present study for comparison (b) J-V characteristics (c) ambient stability.

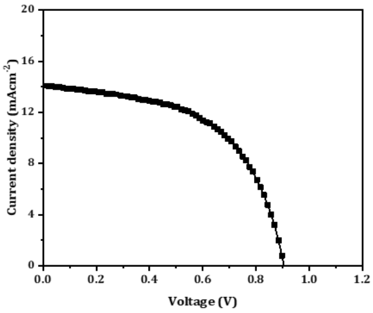

Parameter	Value
V_{OC} (V)	0.905
J_{SC} ($mAcm^{-2}$)	14.12
FF (%)	54.25
PCE (%)	6.91

Figure S21. Dopant-free conventional Spiro-MeOTAD based inorganic solar cells.

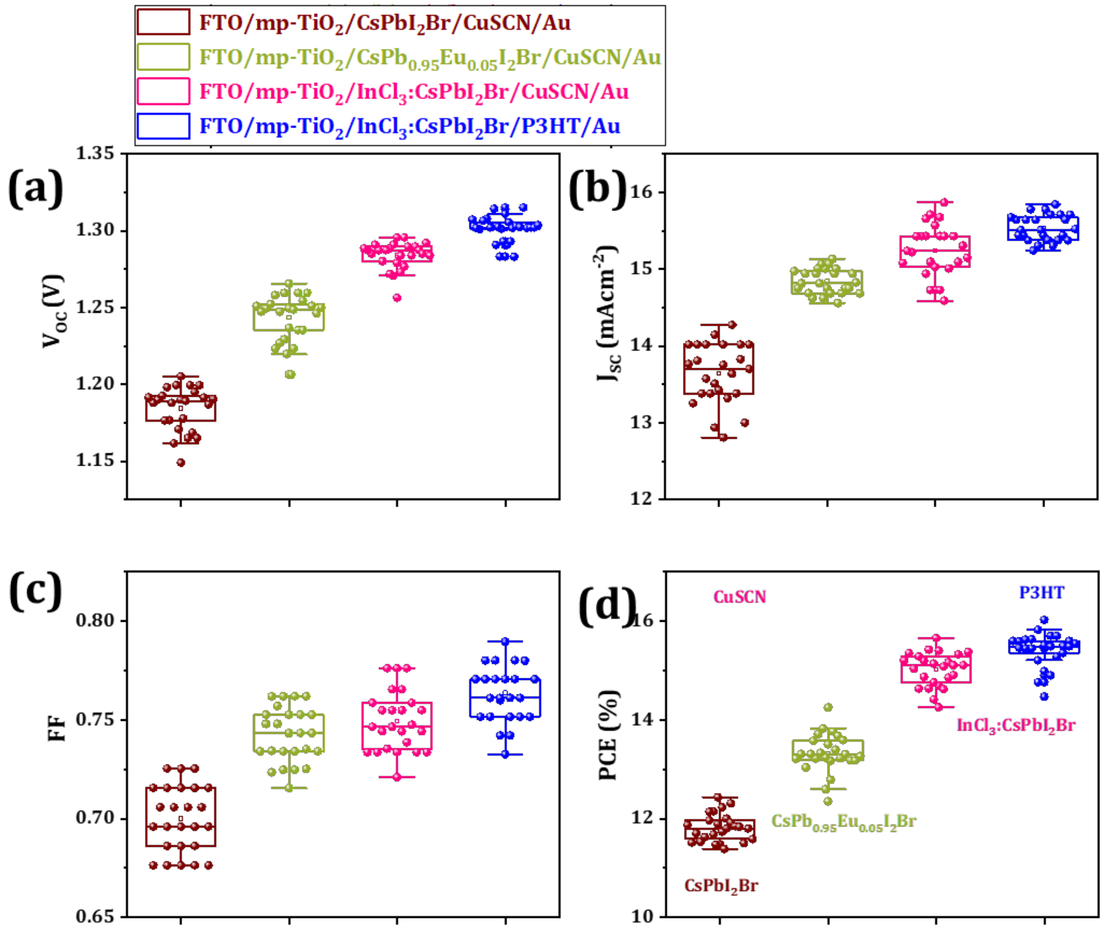

Figure S22. Solar cell parameter distributions for at least 20 perovskite devices for each composition.

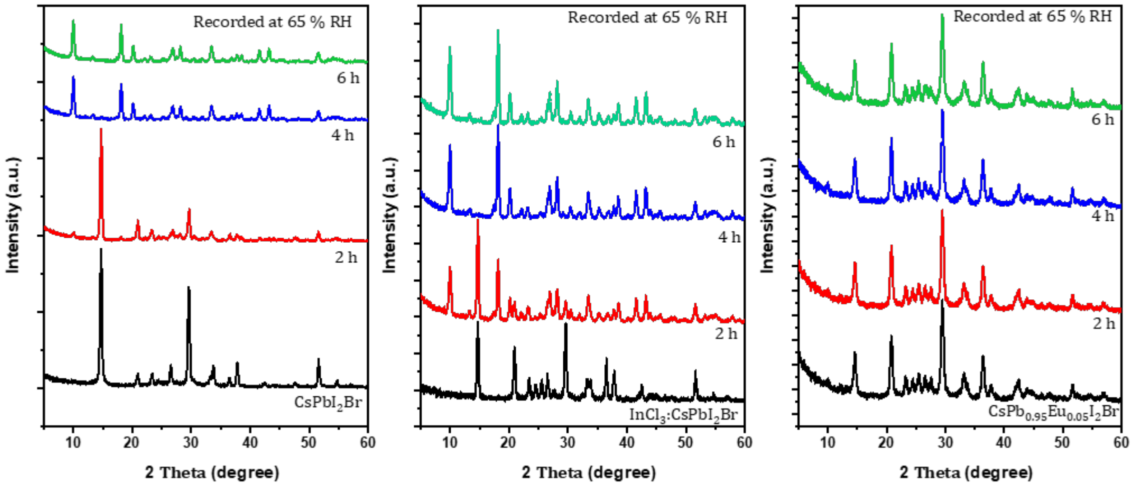

Figure S23. Phase degradation of exposed $CsPbI_2Br$-based perovskite thin films stored under a moisture-rich atmosphere. Note: Humidity was recorded in the XRD chamber at room temperature.

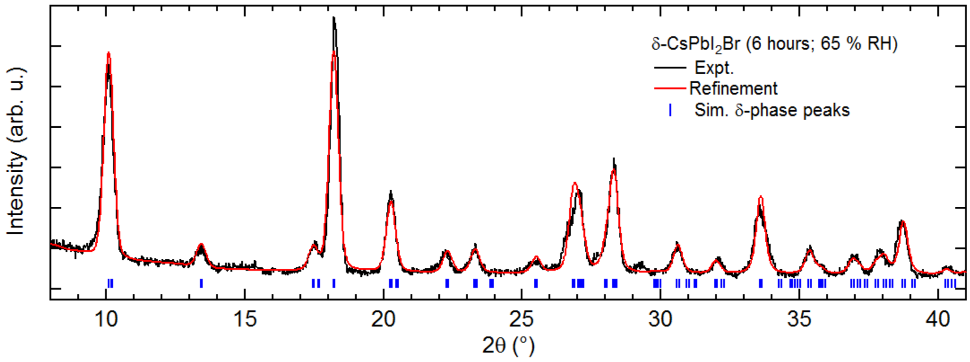

Figure S24. Structural refinement of δ- thin film after 6 hours of ambient storage.

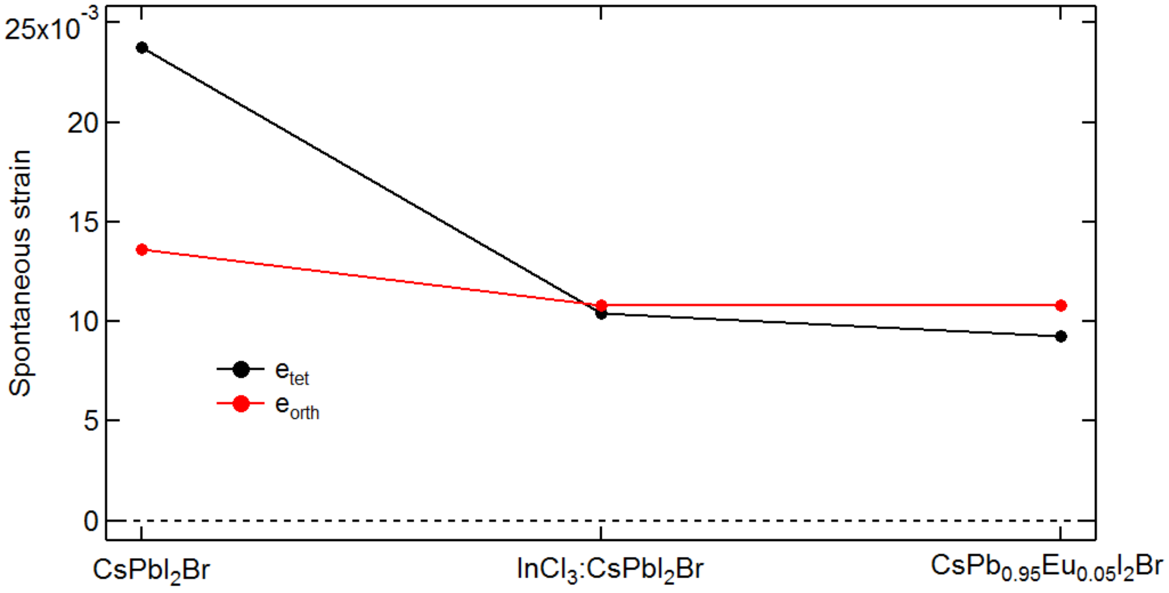

Figure S25. Decoupled orthorhombic (e_{orth}) and tetragonal (e_{tet}) spontaneous strain components for the three different systems under investigation. The joining lines are merely a guide for the eye, with improved long-term stability running left to right.

Supporting Note 6:

The degenerate symmetry-breaking distortions [S16] are thus divided into the tetragonal (e_{tet}) and orthorhombic (e_{orth}) strains, manifesting the β-phase and γ-phase, respectively. These quantities are calculated relative to an undistorted cubic unit cell, a_0, which is estimated by taking the cube root of the normalized unit cell volume. It follows that the spontaneous strain components are defined as: $e_1 = (a - a_0)/a_0, e_2 = (b - a_0)/a_0$ and $e_3 = (c - a_0)/a_0$, where a, b and c are the normalized lattice parameters of the $CsPbI_2Br$-based orthorhombic phase. The separate strain components contributing to the lattice distortions are then given by: $e_{orth} = e_2 - e_1$ and $e_{tet} = (2e_3 - e_2/\sqrt{3}$. A factor of $\sqrt{3}$ is included here to ensure that the two strains are on the same scale.

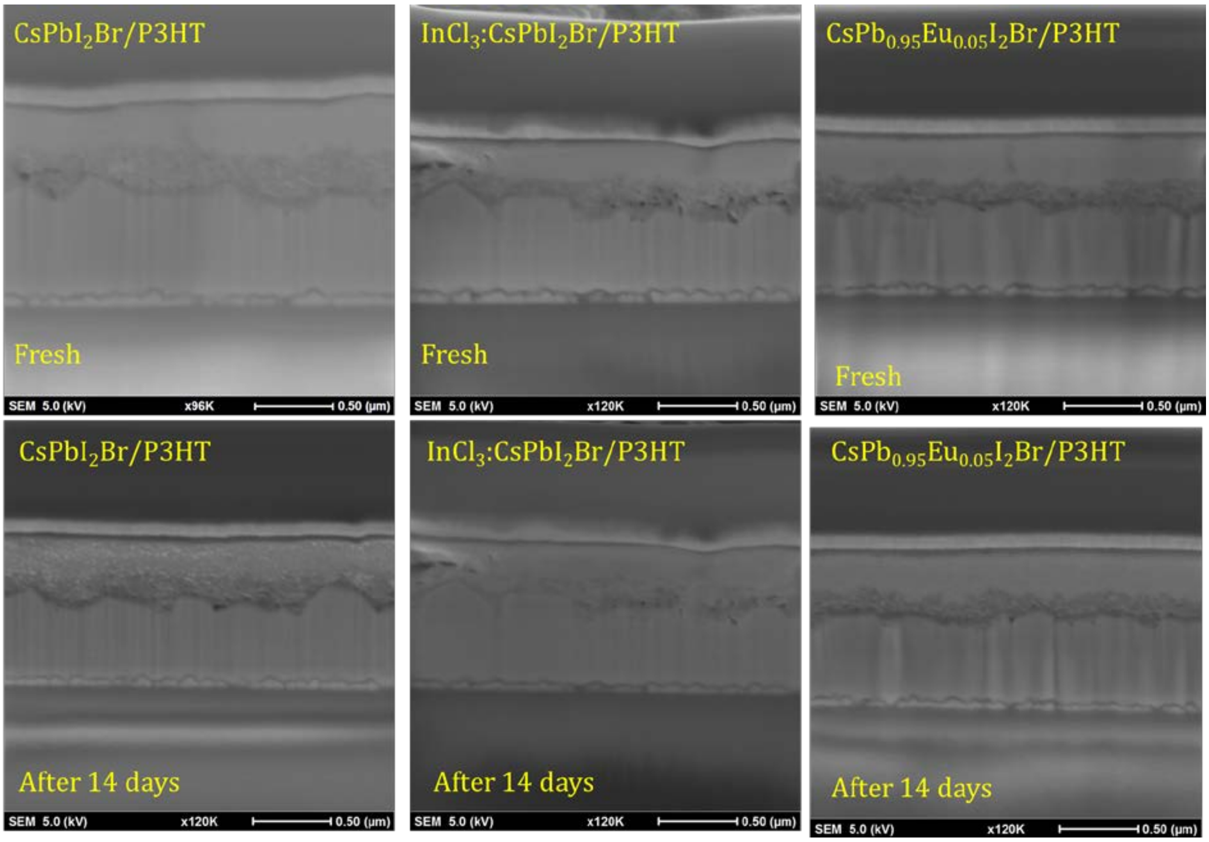

Figure S26. STEM images of $CsPbI_2Br$, $InCl_3$:$CsPbI_2Br$ and $CsPb_{0.95}Eu_{0.05}I_2Br$-based devices recorded after (~10 hours) and 14 days aging.

SUPPORTING REFERENCES:

[S1] Kresse, G. et al. Ab initio molecular dynamics for liquid metals. *Phys. Rev. B: Condens. Matter Mater. Phy.* **1993**, 47, 558–561.

[S2] Kresse, G. et al. From ultrasoft pseudopotentials to the projector augmented-wave method. *Phys. Rev. B: Condens. Matter Mater. Phys.* **1999**, 59, 1758.

[S3] Kresse, G. et al. Efficient iterative schemes for ab initio total-energy calculations using a plane-wave basis set. *Phys. Rev. B: Condens. Matter Mater. Phys.* **1996**, 54, 11169.

[S4] Blöchl, P. et al. Projector augmented-wave method. *Phys. Rev. B.* **1994**, 50, 17953.

[S5] Perdew, J. P.; Burke, K.; Ernzerh, M. Generalized Gradient Approximation Made Simple *Phys. Rev. Lett.,***1997**, *78*, 1396**.**

[S6] Krukau, A. et al. Influence of the exchange screening parameter on the performance of screened hybrid functionals. *J. Chem. Phys.* **2006**, 125, 224106.

[S7] Castelli, I. E.; García-Lastra, J. M.; Thygesen, K. S.; Jacobsen, K. W. Bandgap calculations and trends of organometal halide perovskites. *APL Mater.* **2014**, 2, 081514.

[S8] Mali, S. S., Patil, J. V., Hong C. K. Simultaneous Improved Performance and Thermal Stability of Planar Metal Ion Incorporated $CsPbI_2Br$ All-Inorganic Perovskite Solar Cells Based on MgZnO Nanocrystalline Electron Transporting Layer, *Adv. Energy Mater.* **2020,** 10, 1902708.

[S9] Mali, S. S. *et al.* Fully Air-Processed Dynamic Hot-Air-Assisted M:$CsPbI_2Br$ (M: Eu^{2+}, In^{3+}) for Stable Inorganic Perovskite Solar Cells, Matter, 2020. https://doi.org/10.1016/j.matt.2020.11.008

[S10] de Mello, J.C.; Wittmann, H.F.; Friend, R.H., An improved experimental determination of external photoluminescence quantum efficiency. *Adv. Mater.,* **1997,** 9, 230-232.

[S11] X. Miao, T. Qiu, S. Zhang, H. Ma, Y. Hu, F. Bai, Z. Wu, *J. Mater. Chem. C* **2017,** 5, 4931.

[S12] Robert D. J. Oliver, Yen-Hung Lin, Alexander J. Horn, Chelsea Q. Xia, Jonathan H. Warby, Michael B. Johnston, Alexandra J. Ramadan, Henry J. Snaith, Thermally Stable Passivation Toward High Efficiency Inverted Perovskite Solar Cells. *ACS Energy Lett.* 2020, 5, 3336−3343.

[S13] Stolterfoht, M.; Caprioglio, P.; Wolff, C. M.; Márquez, J. A.; Nordmann, J.; Zhang, S.; Rothhardt, D.; Hörmann, U.; Amir, Y.; Redinger, A.; Kegelmann, L.; Zu, F.; Albrecht, S.; Koch, N.; Kirchartz, T.; Saliba, M.; Unold, T.; Neher, D. The Impact of Energy Alignment and Interfacial Recombination on the Internal and External Open-Circuit Voltage of Perovskite Solar Cells. *Energy Environ. Sci.* **2019**, 12 (9), 2778–2788.

[S14] E.-J. Cho, S.-J. Oh Surface valence transition in trivalent Eu insulating compounds observed by photoelectron spectroscopy, *Physical Review B*, **1999**, 59, 613-616.
[S15] L. Wang, H. Zhou, J. Hu, B. Huang, M. Sun, B. Dong, G. Zheng, Y. Huang, Y. Chen, L. Li, Z. Xu, N. Li, Z. Liu, Q. Chen, L.D. Sun, C.-H. Yan, A Eu^{3+}-Eu^{2+} ion redox shuttle imparts operational durability to Pb-I perovskite solar cells, *Science* **2019,** 363, 265–270.
[S16] Steele, J. A.; Lai, M.; Zhang, Y.; Lin, Z.; Hofkens, J.; Roeffaers, M. B. J.; Yang, P. Phase Transitions and Anion Exchange in All-Inorganic Halide Perovskites. *Acc. Mater. Res.* 2020, 1 (1), 3−15.
